# An experimental test of noncontextuality without unphysical idealizations

**DOI:** 10.1038/ncomms11780

**Published:** 2016-06-13

**Authors:** Michael D. Mazurek, Matthew F. Pusey, Ravi Kunjwal, Kevin J. Resch, Robert W. Spekkens

**Affiliations:** 1 Institute for Quantum Computing, University of Waterloo, Waterloo, Ontario Canada N2L 3G1; 2 Department of Physics & Astronomy, University of Waterloo, Waterloo, Ontario Canada N2L 3G1; 3 Perimeter Institute for Theoretical Physics, 31 Caroline Street North, Waterloo, Ontario Canada N2L 2Y5; 4 Optics & Quantum Information Group, The Institute of Mathematical Sciences, C.I.T Campus, Taramani, Chennai 600 113, India

## Abstract

To make precise the sense in which nature fails to respect classical physics, one requires a formal notion of classicality. Ideally, such a notion should be defined operationally, so that it can be subject to direct experimental test, and it should be applicable in a wide variety of experimental scenarios so that it can cover the breadth of phenomena thought to defy classical understanding. Bell's notion of local causality fulfils the first criterion but not the second. The notion of noncontextuality fulfils the second criterion, but it is a long-standing question whether it can be made to fulfil the first. Previous attempts to test noncontextuality have all assumed idealizations that real experiments cannot achieve, namely noiseless measurements and exact operational equivalences. Here we show how to devise tests that are free of these idealizations. We perform a photonic implementation of one such test, ruling out noncontextual models with high confidence.

## Abstract (web summary)

No noncontextual hidden-variable model can be consistent with quantum theory, but proving such an inconsistency with nature itself is a long-standing problem. Here, the authors devise experimentally-achievable tests of noncontextuality and perform a photonic implementation that rules out such models.

## Graphical abstract

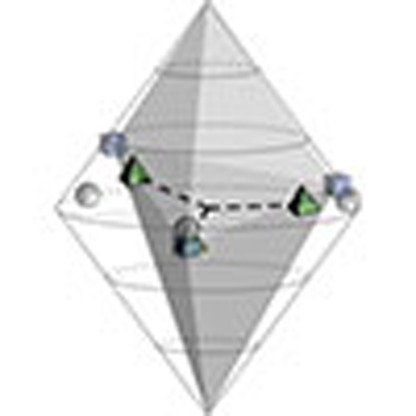

Making precise the manner in which a quantum world differs from a classical one is a surprisingly difficult task. The most successful attempt, due to Bell^1^, shows a conflict between quantum theory and a feature of classical relativistic theories termed local causality, which asserts that no causal influences propagate faster than light. But the latter assumption can only be tested for scenarios wherein there are two or more systems that are space-like separated. And yet few believe that this highly specialized situation is the only point where the quantum departs from the classical. A leading candidate for a notion of nonclassicality with a broader scope is the failure of quantum theory to admit of a noncontextual model, as proven by Kochen and Specker^2^. Recent work has highlighted how this notion lies at the heart of many phenomena that are taken to be distinctly quantum: the fact that quasi-probability representations go negative^3^,^4^, the existence of quantum advantages for cryptography^5^ and for computation^6^,^7^,^8^, and the possibility of anomalous weak values^9^. Consequently, the study of noncontextuality has not only foundational significance but practical applications as well.

An experimental refutation of noncontextuality would demonstrate that the conflict with noncontextual models is not only a feature of quantum theory, but of nature itself, and hence also of any successor to quantum theory. The requirements for such an experimental test, however, have been a subject of much controversy^10^,^11^,^12^,^13^,^14^,^15^,^16^.

A fundamental problem with most proposals for testing noncontextuality^17^,^18^,^19^,^20^,^21^,^22^,^23^,^24^, and experiments performed to date^25^,^26^,^27^,^28^,^29^,^30^,^31^,^32^, is that they seek to test a notion of noncontextuality which posits that measurements have a deterministic response in the noncontextual model. It has been shown that such determinism is only justified under the idealization that measurements are noiseless^33^, which is never satisfied precisely by any real experiment. We refer to this issue as the problem of noisy measurements.

Another critical problem with previous proposals is the fact that the assumption of noncontextuality can only be brought to bear when two measurement events (an event is a measurement and an outcome) are operationally equivalent, which occurs when the two events are assigned exactly the same probability by all preparation procedures^34^; in this case they are said to differ only by the measurement context. In a real experiment, however, one never achieves the ideal of precise operational equivalence. Previous work on testing noncontextuality—including the only experiment to have circumvented the problem of noisy measurements (by focusing on preparations)^5^—has failed to provide a satisfactory account of how the deviation from strict operational equivalence should be accounted for in the interpretation of the results. We term this problem the problem of inexact operational equivalence.

In this work, we solve both of the above problems. We contend with the problem of noisy measurements by devising a test of a generalised notion of noncontextuality, proposed in ref. 34, that allows general measurements to have an indeterministic response while reducing to the traditional notion in the idealized case of projective quantum measurements. For the problem of inexact operational equivalence, whereas some have been led to consider modifying the definition of noncontextuality so that it applies to pairs of procedures that are merely *close* to operationally equivalent^35^,^36^, we circumvent the problem by demonstrating a general technique that appeals to equivalences not among the procedures themselves, but certain convex mixtures thereof. Of course, any judgment of operational equivalence of measurements (preparations) rests on an assumption about which sets of preparations (measurements) are sufficient to establish such equivalence, that is, which sets are tomographically complete. We here assume that the cardinality of a tomographically-complete set of measurements (preparations) for a photon's polarization is three (four), as it is in quantum theory. We collect some experimental evidence for this assumption—another improvement over previous experiments—but the possibility of its failure is the most significant remaining loophole for tests of noncontextuality. For Bell's notion of local causality, the theoretical work of Clauser *et al.*^37^ was critical to enabling an experimental test without unphysical idealizations, e.g., without the perfect anti-correlations presumed in Bell's original proof^1^. Similarly, the theoretical innovations we introduce here make it possible for the first time to subject noncontextuality to an experimental test without the idealizations described above. We report on a quantum-optical experiment of this kind, the results of which rule out noncontextual models with high confidence.

## Results

### A noncontexuality inequality

According to the operational approach proposed in ref. 34, to assume noncontextuality is to assume a constraint on model-construction, namely, that if procedures are statistically equivalent at the operational level then they ought to be statistically equivalent in the underlying model.

Operationally, a system is associated with a set 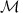 (resp. 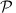) of physically possible measurement (resp. preparation) procedures. An operational theory specifies the possibilities for the conditional probabilities 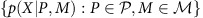 where *X* ranges over the outcomes of measurement *M*. In an ontological model of such a theory, the causal influence of the preparation on the measurement outcome is mediated by the ontic state of the system, that is, a full specification of the system's physical properties. We denote the space of ontic states by Λ. It is presumed that when the preparation *P* is implemented, the ontic state of the system, *λ*∈Λ, is sampled from a probability distribution *μ*(*λ*|*P*), and when the system is subjected to the measurement *M*, the outcome *X* is distributed as *ξ*(*X*|*M*, *λ*). Finally, for the model to reproduce the experimental statistics, we require that


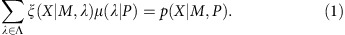


A general discussion of this notion of noncontextuality is provided in Supplementary Note 1, where we also explain how it differs from the notion used in previous experimental tests and why the latter makes unphysical idealizations. This notion can also be understood through the concrete example we consider here (which is based on a construction from Section V of ref. 34).

Suppose there is a measurement procedure, M_*_, that is operationally indistinguishable from a fair coin flip: it always gives a uniformly random outcome regardless of the preparation procedure,


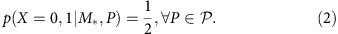


In this case, noncontextuality dictates that in the underlying model, the measurement should also give a uniformly random outcome regardless of the ontic state of the system,


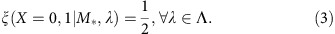


In other words, because M_*_ appears operationally to be just like a coin flip, noncontextuality dictates that physically it must be just like a coin flip.

The second application of noncontextuality is essentially a time-reversed version of the first. Suppose there is a triple of preparation procedures, *P*_1_, *P*_2_ and *P*_3_, that are operationally indistinguishable from one another: no measurement reveals any information about which of these preparations was implemented,


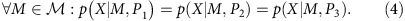


In this case, noncontextuality dictates that in the underlying model, the ontic state of the system does not contain any information about which of these preparation procedures was implemented,


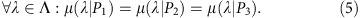


In other words, because it is impossible, operationally, to extract such information, noncontextuality dictates that physically, the information is not present in the system.

Suppose that M_*_ can be realized as a uniform mixture of three other binary-outcome measurements, denoted *M*_1_, *M*_2_ and *M*_3_. That is, one implements M_*_ by uniformly sampling *t*∈{1, 2, 3}, implementing *M*_*t*_, then outputting its outcome as the outcome of M_*_ (ignoring *t* thereafter). Finally, suppose that each preparation *P*_*t*_ can be realized as the equal mixture of two other preparation procedures, denoted *P*_*t*,0_ and *P*_*t*,1_.

Consider implementing *M*_*t*_ on *P*_*t*,*b*_, and consider the average degree of correlation between the measurement outcome *X* and the preparation variable *b*:


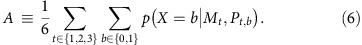


We now show that noncontextuality implies a nontrivial bound on *A*.

The proof is by contradiction. In order to have perfect correlation on average, we require perfect correlation in each term, which implies that for all ontic states *λ* assigned nonzero probability by *P*_*t*,*b*_, the measurement *M*_*t*_ must respond deterministically with the *X*=*b* outcome. Given that *P*_*t*_ is an equal mixture of *P*_*t*,0_ and *P*_*t*,1_, it follows that for all ontic states *λ* assigned nonzero probability by *P*_*t*_, the measurement *M*_*t*_ must have a deterministic response, i.e., *ξ*(*X*=*b*|*M*_*t*_, *λ*)∈{0, 1}.

But equation (5) (which follows from the assumption of noncontextuality) asserts that the preparations *P*_1_, *P*_2_ and *P*_3_ must assign nonzero probability to precisely the same set of ontic states. Therefore, to achieve perfect correlation on average, each measurement must respond deterministically to all the ontic states in this set.

Now note that by the definition of M_*_, the probability of its outcome *X*=*b* is 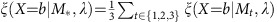. But then equation (3) (which follows from the assumption of noncontextuality) says


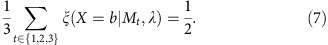


For each deterministic assignment of values, (*ξ*(*X*=*b*|*M*_1_, *λ*), *ξ*(*X*=*b*|*M*_2_, *λ*), *ξ*(*X*=*b*|*M*_3_, *λ*))∈{(0, 0, 0), (0, 0, 1), …, (1, 1, 1)}, the constraint of equation (7) is violated. It follows, therefore, that for a given *λ*, one of *M*_1_, *M*_2_ or *M*_3_ must fail to have a deterministic response, contradicting the requirement for perfect correlation on average. This concludes the proof.

The precise (i.e., tight) bound is


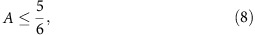


as we demonstrate in Supplementary Figs 1–3, Supplementary Tables 1 and 2, and Supplementary Note 2. This is our noncontextuality inequality.

### Quantum violation of the inequality

Quantum theory predicts there is a set of preparations and measurements on a qubit having the supposed properties and achieving *A*=1, the logical maximum. Take the *M*_*t*_ to be represented by the observables ***σ***·***n***_*t*_ where **σ** is the vector of Pauli operators and the unit vectors {***n***_1_, ***n***_2_, ***n***_3_} are separated by 120° in the ***x***−***z*** plane of the Bloch sphere of qubit states^38^. The *P*_*t*,*b*_ are the eigenstates of these observables, where we associate the positive eigenstate |+***n***_*t*_〉〈+***n***_*t*_| with *b*=0. To see that the statistical equivalence of equation (2) is satisfied, it suffices to note that


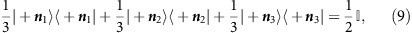


and to recall that for any density operator *ρ*, 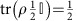. To see that the statistical equivalence of equation (4) is satisfied, it suffices to note that for all pairs *t*, *t*′∈{1, 2, 3},


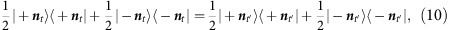


which asserts that the average density operator for each value of *t* is the same, and therefore leads to precisely the same statistics for all measurements. Finally, it is clear that the outcome of the measurement of ***σ***·***n***_*t*_ is necessarily perfectly correlated with whether the state was |+***n***_*t*_〉〈+***n***_*t*_| or |−***n***_*t*_〉〈−***n***_*t*_|, so that *A*=1.

These quantum measurements and preparations are what we seek to implement experimentally, so we refer to them as ideal, and denote them by 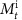 and 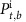.

Note that our noncontextuality inequality can accommodate noise in both the measurements and the preparations, up to the point where the average of *p*(*X*=*b*|*M*_*t*_, *P*_*t*,*b*_) drops below 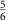. It is in this sense that our inequality does not presume the idealization of noiseless measurements.

### Contending with the lack of exact operational equivalence

The actual preparations and measurements in the experiment, which we call the primary procedures and denote by 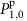, 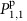, 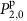, 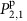, 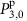, 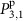 and 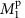, 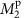, 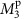, almost certainly deviate from the ideal versions and consequently their mixtures, that is, 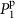, 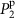, 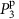 and 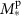, fail to achieve strict equality in equations (2) and (4).

We solve this problem as follows. From the outcome probabilities on the six primary preparations, one can infer the outcome probabilities on the entire family of probabilistic mixtures of these. It is possible to find within this family many sets of six preparations, 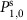, 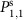, 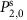, 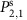, 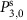, 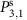, which define mixed preparations 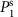, 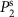, 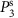 that satisfy the operational equivalences of equation (4) exactly. We call the 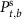 secondary preparations. We can define secondary measurements 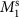, 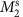, 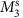 and their uniform mixture 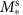 in a similar fashion. The essence of our approach, then, is to identify such secondary sets of procedures and use these to calculate *A*. If quantum theory correctly models our experiment, then we expect to get a value of *A* close to 1 if and only if we can find suitable secondary procedures that are close to the ideal versions.

To test the hypothesis of noncontextuality, one must allow for the possibility that the experimental procedures do not admit of a quantum model. Nonetheless, for pedagogical purposes, we will first provide the details of how one would construct the secondary sets under the assumption that all the experimental procedures do admit of a quantum model.

**Figure 1:**
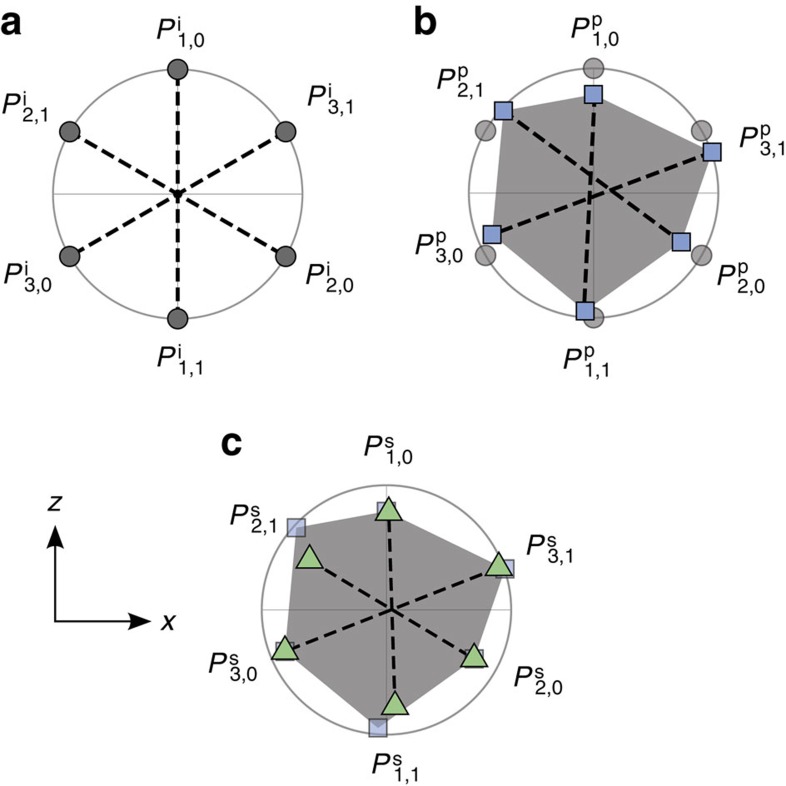
Solution to the problem of inexact operational equivalences. Here, we illustrate our solution for the case of preparations under the simplifying assumption that these are confined to the ***x***−***z*** plane of the Bloch sphere. For a given pair, *P*_*t*,0_ and *P*_*t*,1_, the midpoint along the line connecting the corresponding points represents their equal mixture, *P*_*t*_. (**a**) The target preparations 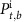, with the coincidence of the midpoints of the three lines illustrating that they satisfy the operational equivalence (4) exactly. (**b**) Illustration of how errors in the experiment (exaggerated in magnitude) will imply that the realized preparations 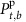 (termed primary) will deviate from the ideal. The lines indicate that not only do these preparations fail to satify the operational equivalence (4), but since the three lines do not all meet at the same point, no mixtures of the 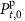 and 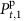 can be found at a single point independent of *t*. The set of preparations corresponding to probabilistic mixtures of the 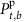 are depicted by the grey region. (**c**) Secondary preparations 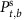 have been chosen from this grey region, with the coincidence of the midpoints of the three lines indicating that the operational equivalence (4) has been restored. Note that we require only that the mixtures of the three pairs of preparations be the same, not that they correspond to the completely mixed state.

In Fig. 1, we describe the construction of secondary preparations in a simplified example of six density operators that deviate from the ideal states only within the ***x***−***z*** plane of the Bloch sphere.

In practice, the six density operators realized in the experiment will not quite lie in a plane. We use the same idea to contend with this, but with one refinement: we supplement our set of ideal preparations with two additional ones, denoted 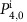 and 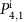 corresponding to the two eigenstates of ***σ***·***y***. The two procedures that are actually realized in the experiment are denoted 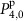 and 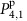 and are considered supplements to the primary set. We then search for our six secondary preparations among the probabilistic mixtures of this supplemented set of primaries rather than among the probabilistic mixtures of the original set. Without this refinement, it can happen that one cannot find six secondary preparations that are close to the ideal versions, as we explain in Supplementary Note 3.

The scheme for defining secondary measurement procedures is also described in Supplementary Fig. 4 and Supplementary Note 3. Analogously to the case of preparations, one contends with deviations from the plane by supplementing the ideal set with the observable ***σ***·***y***.

Note that in order to identify which density operators have been realized in an experiment, the set of measurements must be complete for state tomography^39^. Similarly, to identify which sets of effects have been realized, the set of preparations must be complete for measurement tomography^40^. However, the original ideal sets fail to be tomographically complete because they are restricted to a plane of the Bloch sphere, and an effective way to complete them is to add the observable ***σ***·***y*** to the measurements and its eigenstates to the preparations. Therefore, even if we did not already need to supplement these ideal sets for the purpose of providing greater leeway in the construction of the secondary procedures, we would be forced to do so in order to ensure that one can achieve full tomography.

The relevant procedure here is not quite state tomography in the usual sense, since we want to allow for systematic errors in the measurements as well as the preparations. Hence the task^41^,^42^ is to find a set of qubit density operators, *ρ*_*t*,*b*_, and POVMs, {*E*_*X*|*t*_}, that together make the measured data as likely as possible (we cannot expect tr(*ρ*_*t*,*b*_*E*_*X*|*t*_) to match the measured relative frequencies exactly due to the finite number of experimental runs).

To analyze our data in a manner that does not prejudice which model—noncontextual, quantum, or otherwise—does justice to it, we must search for representations of the preparations and measurements not amongst density operators and sets of effects, but rather their more abstract counterparts in the formalism of generalised probabilistic theories^43^,^44^ (GPTs), called generalised states and effects. The assumption that the system is a qubit is replaced by the strictly weaker assumption that three two-outcome measurements are tomographically complete. (In GPTs, a set of measurements are called tomographically complete if their statistics suffice to determine the state.) We take these states and effects as estimates of our primary preparations and measurements, and we define our estimate of the secondary procedures in terms of these, which in turn are used to calculate our estimate for *A*. We explain how the raw data is fit to a set of generalised states and effects in Supplementary Note 4. We characterize the quality of this fit with a *χ*^2^ test.

### Experiment

**Figure 2:**
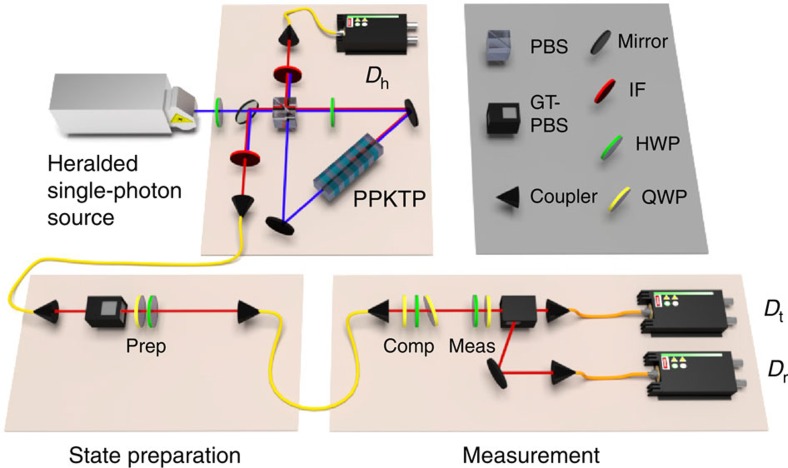
The experimental setup. Polarization-separable photon pairs are created via parametric downconversion, and detection of a photon at detector *D*_h_ heralds the presence of a single photon. The polarization state of this photon is prepared with a polarizer and two waveplates (Prep). A single-mode fibre is a spatial filter that decouples beam deflections caused by the state-preparation and measurement waveplates from the coupling efficiency into the detectors. Three waveplates (Comp) are set to undo the polarization rotation caused by the fibre. Two waveplates (Meas), a polarizing beamsplitter, and detectors *D*_r_ and *D*_t_ perform a two-outcome measurement on the state. PPKTP, periodically-poled potassium titanyl phosphate; PBS, polarizing beamsplitter; GT-PBS, Glan-Taylor polarizing beamsplitter; IF, interference filter; HWP, half-waveplate; QWP, quarter-waveplate.

We use the polarization of single photons to test our noncontextuality inequality. The set-up, shown in Fig. 2, consists of a heralded single-photon source^45^,^46^,^47^, polarization-state preparation and polarization measurement. We generate photons using spontaneous parametric downconversion and prepare eight polarization states using a polarizer followed by a quarter-wave plate (QWP) and half-wave plate (HWP). The four polarization measurements are performed using a HWP, QWP and polarizing beamsplitter. Photons are counted after the beamsplitter and the counts are taken to be fair samples of the true probabilities for obtaining each outcome for every preparation-measurement pair. Since the orientations of the preparation waveplates lead to small deflections of the beam, some information about the preparation gets encoded spatially, and similarly the measurement waveplates create sensitivity to spatial information; coupling the beam into the single-mode fibre connecting the state-preparation and measurement stages of the experiment removes sensitivity to these effects. For a single experimental run we implement each preparation-measurement pair for 4 s (approximately 10^5^ counts). We performed 100 such runs.

**Figure 3:**
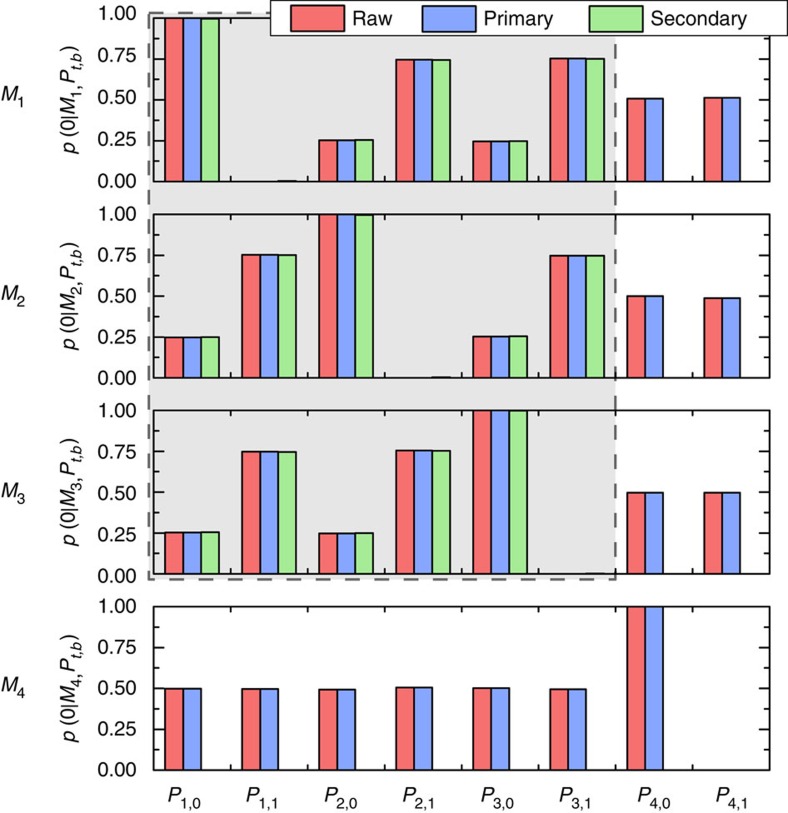
Outcome probabilities for each measurement-preparation pair. For each such pair, we report the probability of obtaining outcome 0 in the measurement. Red bars are relative frequencies calculated from the raw counts, blue bars are our estimates of the outcome probabilities of the primary measurements on the primary preparations obtained from a best-fit of the raw data, and green bars are our estimates of the outcome probabilities of the secondary measurements on the secondary preparations. The shaded grey background highlights the measurements and preparations for which secondary procedures were found. Error bars are not visible on this scale, neither are discrepancies between the obtained probabilities and the ideal values thereof, which are at most 0.013; statistical error due to Poissonian count statistics is at most 0.002.

**Figure 4:**
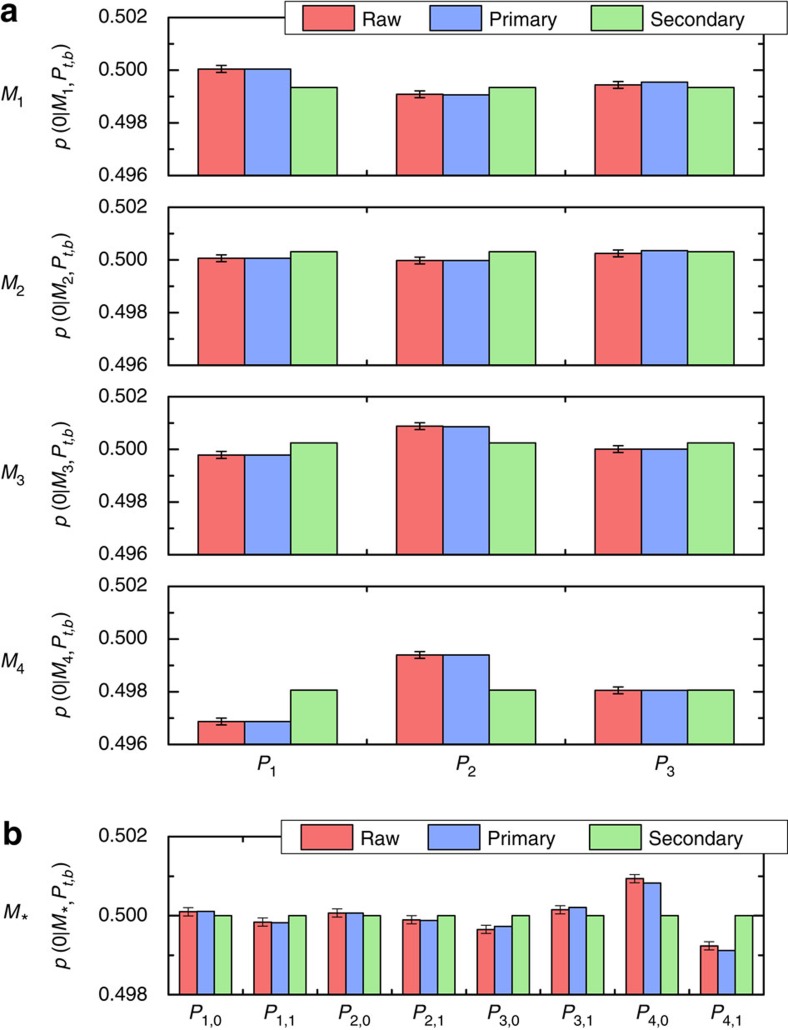
Verifying the requisite operational equivalences. Operational statistics for raw, primary, and secondary preparations and measurements, averaged over 100 experimental runs. (**a**) The probabilities of the primary measurements (blue bars) differ depending on which of the three mixed preparations 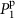, 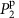, and 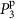 are measured. These probabilities are within error of the raw data (red bars), indicating that a generalised probabilistic theory in which three two-outcome measurements are tomographically complete fits the data well. Probabilities for primary measurements on the secondary preparations (green bars) are independent of the preparation, hence the secondary preparations satisfy equation (4). Note that one expects these probabilities to deviate from 0.5. In the example of Fig. 1c, this corresponds to the fact that the intersection of the lines is not the completely mixed state. (**b**) Outcome probabilities of measurement M_*_ on the eight preparations. Red bars are raw data, blue bars are the measurement 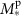 on the primary preparations, and green bars are 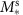 on the primary preparations. Regardless of the input state, 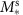 returns outcome 0 with probability 0.5, hence it is operationally indistinguishable from a fair-coin flip (equation (2)). Error bars in all plots are calculated assuming Poissonian count statistics.

Preparations are represented by vectors of raw data specifying the relative frequencies of outcomes for each measurement, uncertainties on which are calculated assuming Poissonian uncertainty in the photon counts. For each run, the raw data is fit to a set of states and effects in a GPT in which three binary-outcome measurements are tomographically complete. This is done using a total weighted least-squares method^48^,^49^. The average *χ*^2^ over the 100 runs is 3.9±0.3, agreeing with the expected value of 4, and indicating that the model fits the data well (see Supplementary Note 4, Supplementary Data 1 and 2, and Supplementary Software 1). The fit returns a 4 × 8 matrix that serves to define the 8 GPT states and 4 GPT effects, which are our estimates of the primary preparations and measurements. The column of this matrix associated to the *t*,*b* preparation, which we denote 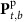, specifies our estimate of the probabilities assigned by the primary preparation 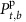 to outcome ‘0' of each of the primary measurements. The raw and primary data are compared in Fig. 3. The probabilities are indistinguishable on this scale. We plot the probabilities for *P*_1_, *P*_2_, and *P*_3_ in Fig. 4a on a much finer scale. We then see that the primary data are within error of the raw data, as expected given the high quality of the fit to the GPT. However, the operational equivalences of equations (2) and (4) are not satisfied by our estimates of the primary preparations and measurements, illustrating the need for secondary procedures.

We define the six secondary preparations as probabilistic mixtures of the eight primaries: 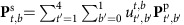, where the 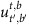 are the weights in the mixture. We maximize 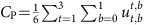 over valid 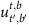 subject to the constraint of equation (4), that is, 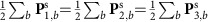 (a linear program). A high value of *C*_P_ ensures that each of the six secondary preparations is close to its corresponding primary. Averaging over 100 runs, we find *C*_P_=0.9969±0.0001, close to the maximum of 1. An analogous linear program to select secondary measurements yields similar results. Supplementary Tables 3 and 4 display the weights that define each secondary preparation and measurement, averaged over 100 runs. Figure 3 also displays the outcome probabilities for the secondary procedures, confirming that they are close to ideal. Figure 4 demonstrates how our construction enforces the operational equivalences.

**Figure 5:**
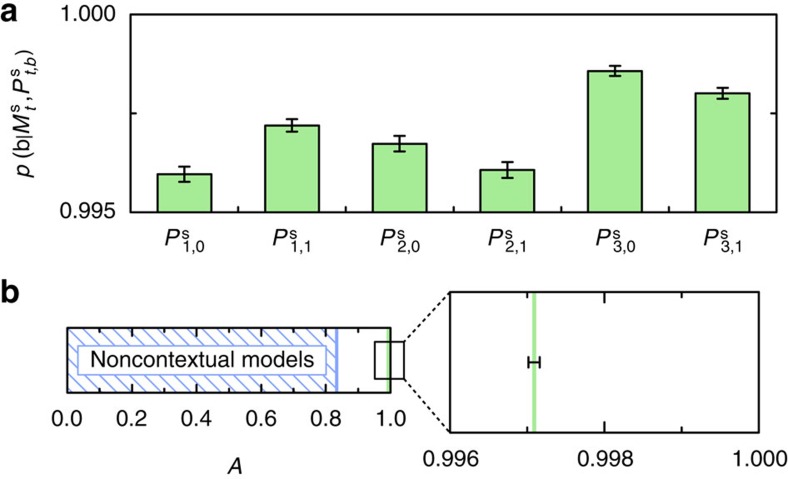
Violation of the noncontextuality inequality. (**a**) Values of the six degrees of correlation in equation (8), averaged over 100 experimental runs. (**b**) Average measured value for *A* contrasted with the noncontextual bound *A*=5/6. We find *A*=0.99709±0.00007, which violates the noncontextual bound by 2300*σ*. Error bars in both plots represent the standard deviation in the average of the measured values over the 100 experimental runs.

We analyzed each experimental run separately and found the degree of correlation 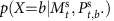 for each value of *t* and *b*. The averages over the 100 runs are shown in Fig. 5a and are all in excess of 0.995. Averaging over *t* and *b* yields an experimental value *A*=0.99709±0.00007, which violates the noncontextual bound of 5/6≈0.833 by 2300*σ* (Fig. 5b).

## Discussion

Using the techniques described here, it is possible to convert proofs of the failure of noncontextuality in quantum theory into experimental tests of noncontextuality that are robust to noise and experimental imprecisions^50^,^51^. For any phenomenon, therefore, one can determine which of its operational features are genuinely nonclassical. This is likely to have applications for scientific fields wherein quantum effects are important and for developing novel quantum technologies. The definition of operational equivalence of preparations (measurements) required them to be statistically equivalent relative to a tomographically complete set of measurements (preparations). There are two examples of how the assumption of tomographic completeness is expected not to hold exactly in our experiment, even if one grants the correctness of quantum theory. First, our source produces a small multi-photon component. We measure the *g*^(2)^(0) of our source^52^ to be 0.0105±0.0001 and from this we estimate the ratio of heralded detection events caused by multiple photons to those caused by single photons to be 1:4,000. Regardless of the value of *A* that one presumes for multi-photon events, one can infer that the value of *A* we would have achieved had the source been purely single-photon could have been less than the value given above by at most 10^−6^, a difference that does not affect our conclusions. We also expect the assumption to not hold exactly because of the inevitable coupling of the polarization into the spatial degree of freedom of the photon, which could be caused, for example, by a wedge in a waveplate. Indeed, we found that if the spatial filter was omitted from the experiment, our fitting routine returned large *χ*^2^ values, which we attributed to the fact that different angles of the waveplates led to different deflections of the beam. A more abstract worry is that nature might conflict with the assumption (and prediction of quantum theory) that three independent binary-outcome measurements are tomographically complete for the polarization of a photon. Our experiment has provided evidence in favour of the assumption insofar as we have fit data from four measurements to a theory where three are tomographically complete and found a good *χ*^2^ value for the fit. One can imagine accumulating much more evidence of this sort, but it is difficult to see how any experiment could conclusively vindicate the assumption, given that one can never test all possible measurements. This, therefore, represents the most significant loophole in experimental tests of noncontextuality, and new ideas for how one might seal it or circumvent it represent the new frontier for improving such tests.

## Methods

### Preparation procedure

A 20-mW diode laser with a wavelength of 404.7 nm produces photon pairs, one horizontally polarized and the other vertically polarized, via spontaneous parametric down-conversion in a 20-mm type-II PPKTP crystal. The downconversion crystal is inside a Sagnac loop and the pump laser is polarized vertically to ensure it only travels counter-clockwise around the loop. Photon pairs are separated at a polarizing beamsplitter and coupled into two single-mode fibres (SMFs). Vertically-polarized photons are detected immediately at detector *D*_h_, heralding the presence of the horizontally-polarized signal photons which emerge from the SMF and pass through a state-preparation stage before they are measured. Herald photons were detected at a rate of 400 kHz. Signal photons emerge from the fibre and pass through a Glan-Taylor polarizing beamsplitter (GT-PBS) which transmits vertically-polarized light. Polarization controllers in the fibre maximize the number of photons which pass through the beamsplitter. A quarter- and half-waveplate set the polarization of the signal photons to one of eight states.

### Spatial mode filter

A single-mode fibre acts as a spatial mode filter. This filter ensures that information about the angles of the state-preparation waveplates cannot be encoded in the spatial mode of the photons, and that our measurement procedures do not have a response that depends on the spatial mode, but only on polarization as intended. The SMF induces a fixed polarization rotation, so a set of three compensation waveplates are included after the SMF to undo this rotation. It follows that the preparation-measurement pairs implemented in our experiment are in fact a rotated version of the ideal preparation and a similarly-rotated version of the ideal measurement. Such a fixed rotation, however, does not impact any of our analysis.

### Measurement procedure

Measurements are performed in four bases, set by a half- and quarter-waveplate. A second GT-PBS splits the light, and both output ports are detected. Due to differences in the coupling and detection efficiencies in each path after the beamsplitter, each measurement consists of two parts. First, the waveplates are aligned such that states corresponding to outcome ‘0' are transmitted by the GT-PBS, and the number of heralded photons detected in a two-second window is recorded for each port. Second, the waveplate angles are changed in such a way as to invert the outcomes, so the detector in the reflected port corresponds to outcome ‘0' and heralded photons are detected for another two seconds. The counts are added together and the probability for outcome ‘0' is calculated by dividing the number of detections corresponding to outcome ‘0' by the total number of detection events in the four-second window. The single-photon detection rate at detectors *D*_r_ and *D*_t_ depends on the measurement settings. In the transmissive and reflective ports of the measurement GT-PBS photons were detected at maximum rates of 330 and 250 kHz, respectively. Coincident detection events between herald photons and the transmissive and reflective ports of the measurement GT-PBS were up to 22 and 16 kHz, respectively.

### Code availability

The authors declare that the data-analysis code supporting the findings of this study are available within the article's Supplementary Information files (Supplementary Software 1).

### Data availability

The authors declare that the data supporting the findings of this study are available within the article and its Supplementary Files (Supplementary Data 1 and 2).

## Additional information

**How to cite this article:** Mazurek, M. D. *et al.* An experimental test of noncontextuality without unphysical idealizations. *Nat. Commun.* 7:11780 doi: 10.1038/ncomms11780 (2016).

## Supplementary Material

Supplementary Information Supplementary Figures 1-4, Supplementary Tables 1-4, Supplementary Notes 1-4 and Supplementary References

Supplementary Data 1 Microsoft Excel file containing coincidence counts for each preparation-measurement pair, for each of 100 experimental runs.

Supplementary Data 2 Microsoft Excel file containing coincidence counts for each preparation-measurement pair, integrated over all 100 experimental runs.

Supplementary Software 1 Mathematica notebook which performs the fitting routine (to find characterizations of the primary preparation and measurement procedures given the raw data) and linear optimization (to find characterizations of the secondary procedures given the primary ones) for each of the 100 experimental runs.
