# Supplementary material for: An experimental test of noncontextuality without unphysical idealizations
Source: Nat Commun. 2016 Jun 13;7:ncomms11780. doi: 10.1038/ncomms11780 (PMC4909951; doi:10.1038/ncomms11780)
Supplement: Supplementary Information — Supplementary Figures 1-4, Supplementary Tables 1-4, Supplementary Notes 1-4 and Supplementary References [file ncomms11780-s1.pdf]

SUPPLEMENTARY FIGURES

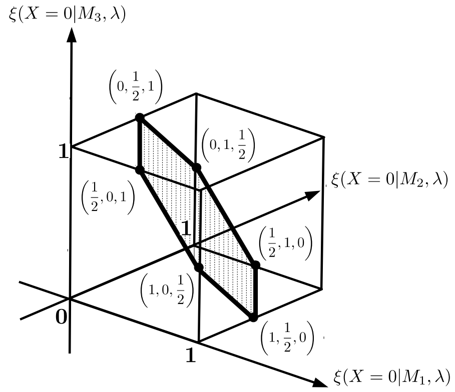

Supplementary Figure 1: Possible values of noncontextual measurement response functions. Possible values of the vector of response functions $(\xi(X{=}0|M_1,\lambda),\xi(X{=}0|M_2,\lambda),\xi(X{=}0|M_3,\lambda))$. The range of each response function is $[0,1]$, constraining the vectors to lie inside the unit cube. This fact, along with the linear constraint in Supplementary Equation (17), constrains the vectors to the shaded polygon.

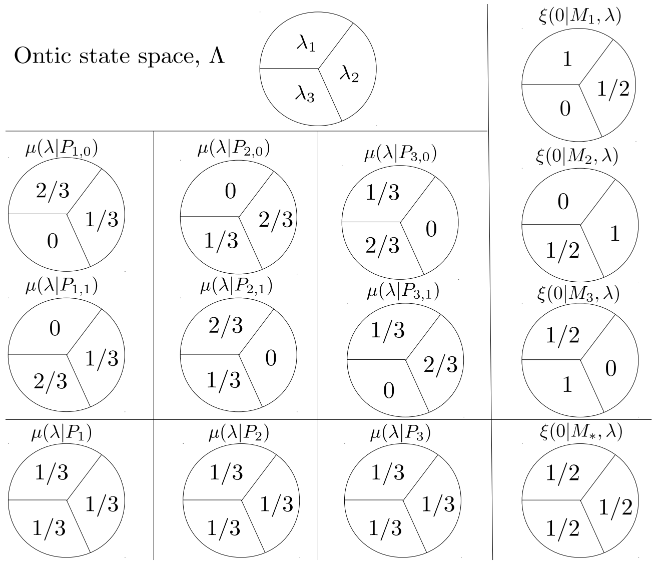

Supplementary Figure 2: Demonstration that the bound of our noncontextuality inequality is tight. The figure displays a noncontextual ontological model that saturates the noncontextual bound of our inequality.

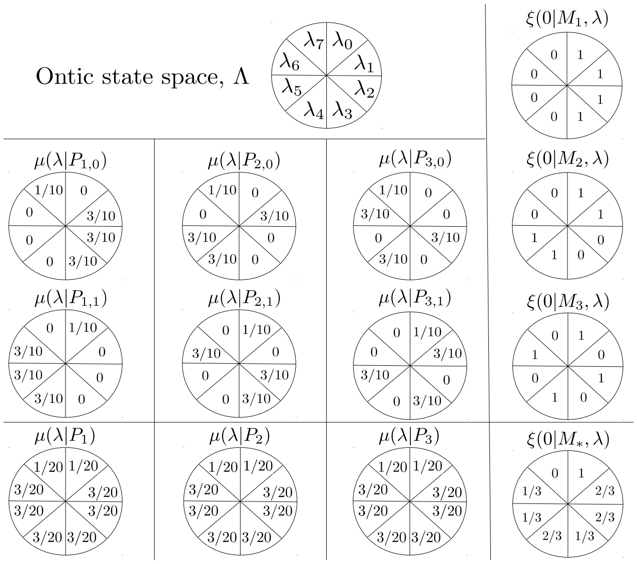

Supplementary Figure 3: A measurement-contextual ontological model violating our inequality. The assumption of measurement noncontextuality is necessary to derive the precise bound in our noncontextuality inequality. The figure presents an ontological model that is preparation noncontextual but measurement contextual and that violates our inequality by achieving a value of $A = 9/10$.

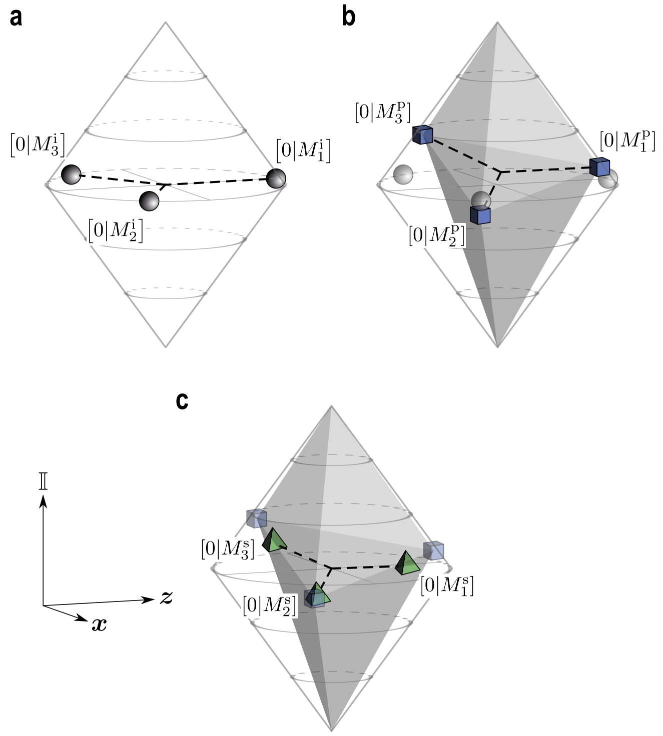

Supplementary Figure 4: Enforcing operational equivalence for measurements. A depiction of the construction of secondary measurements from primary ones in the simplified case where the component along $\boldsymbol{y}$ is zero. For each measurement, we specify the point corresponding to the Bloch representation of its first outcome. These are labelled $[0|M_1]$, $[0|M_2]$ and $[0|M_3]$. The equal mixture of these three, labelled $[0|M_*]$, is the centroid of these three points, i.e. the point equidistant from all three. **a**, The ideal measurements $[0|M_t^{\mathrm{i}}]$ with centroid at $\mathbb{I}/2$, illustrating that the operational equivalence of Equation (2) from the main text is satisfied exactly. **b**, Errors in the experiment (exaggerated) will imply that the realized measurements $[0|M_t^{\mathrm{p}}]$ (termed primary) will deviate from the ideal, and their centroid deviates from $\mathbb{I}/2$. The family of points corresponding to probabilistic mixtures of the $[0|M_t^{\mathrm{p}}]$ and the observables 0 and $\mathbb{I}$ are depicted by the grey region. (For clarity, we have not depicted the outcome-flipped versions of the three primary measurements, and have not included them in the probabilistic mixtures. As we note in the text, such a restriction still allows for a good construction.) **c**, The secondary measurements M_t^{s} that have been chosen from this grey region. They are chosen such that their centroid is at $\mathbb{I}/2$, restoring the operational equivalence of Equation (2) from the main text.

SUPPLEMENTARY TABLES

	$[0\|M_1]$	$[0\|M_2]$	$[0\|M_3]$	$[0\|M_*]$
$P_{1,0}$	5/6	1/3	1/3	1/2
$P_{1,1}$	1/6	2/3	2/3	1/2
$P_{2,0}$	1/3	5/6	1/3	1/2
$P_{2,1}$	2/3	1/6	2/3	1/2
$P_{3,0}$	1/3	1/3	5/6	1/2
$P_{3,1}$	2/3	2/3	1/6	1/2
P_1	1/2	1/2	1/2	1/2
P_2	1/2	1/2	1/2	1/2
P_3	1/2	1/2	1/2	1/2

Supplementary Table 1: Operational statistics of a noncontextual ontological model that saturates our inequality. Operational statistics from the noncontextual ontological model of Supplementary Figure 2, achieving $A = 5/6$. The shaded cells correspond to the ones relevant for calculating A.

	$[0\|M_1]$	$[0\|M_2]$	$[0\|M_3]$	$[0\|M_*]$
$P_{1,0}$	9/10	3/10	3/10	1/2
$P_{1,1}$	1/10	7/10	7/10	1/2
$P_{2,0}$	3/10	9/10	3/10	1/2
$P_{2,1}$	7/10	1/10	7/10	1/2
$P_{3,0}$	3/10	3/10	9/10	1/2
$P_{3,1}$	7/10	7/10	1/10	1/2
P_1	1/2	1/2	1/2	1/2
P_2	1/2	1/2	1/2	1/2
P_3	1/2	1/2	1/2	1/2

Supplementary Table 2: Operational statistics of a measurement-contextual ontological model that violates our inequality. Operational statistics from the preparation noncontextual and measurement contextual ontological model of Supplementary Figure 3, achieving $A = 9/10$. The shaded cells correspond to the ones relevant for calculating A.

	$P^{\rm p}_{1,0}$	$P^{\rm p}_{1,1}$	$P^{\rm p}_{2,0}$	$P^{\rm p}_{2,1}$	$P^{\rm p}_{3,0}$	$P^{\rm p}_{3,1}$	$P^{\rm p}_{4,0}$	$P^{\rm p}_{4,1}$
$P^{\rm s}_{1,0}$	0.99483	0.00023	0.00029	0.00092	0.00016	0.00031	0.00324	0.00003
$P^{\rm s}_{1,1}$	0.00002	0.99791	0.00014	0.00026	0.00006	0.00005	0.00154	0.00002
$P^{\rm s}_{2,0}$	0.00065	0.00008	0.99684	0.00003	0.00001	0.00029	0.00002	0.00208
$P^{\rm s}_{2,1}$	0.00134	0.00015	0.00009	0.99482	0.00008	0.00028	0.00000	0.00323
$P^{\rm s}_{3,0}$	0.00008	0.00023	0.00011	0.00000	0.99883	0.00004	0.00044	0.00027
$P^{\rm s}_{3,1}$	0.00011	0.00023	0.00022	0.00016	0.00016	0.99803	0.00050	0.00061

Supplementary Table 3: Values of the weights used to define each secondary preparation procedure. Each of the six secondary preparation procedures, denoted $P^{\rm s}_{t,b}$ where $t \in \{1,2,3\}, b \in \{0,1\}$ (the rows), is a probabilistic mixture of the eight primary preparation procedures, denoted $P^{\rm p}_{t',b'}$ where $t' \in \{1,2,3,4\}, b' \in \{0,1\}$ (the columns). The table presents the weights appearing in each such mixture, denoted $u^{t,b}_{t',b'}$ in the main text. These are determined numerically by maximizing the function $C_{\rm P} = \frac{1}{6}\sum_{t=1}^{3}\sum_{b=0}^{1} u^{t,b}_{t,b}$ (the average of the weights appearing in the shaded cells), which quantifies the closeness of the secondary procedures to the primary ones, subject to the constraint of operational equivalence of the uniform mixtures of $P^{\rm s}_{t,0}$ and $P^{\rm s}_{t,1}$ for $t \in \{1,2,3\}$. The values presented are averages over 100 runs.

	$[0\|M^{\rm p}_1]$	$[0\|M^{\rm p}_2]$	$[0\|M^{\rm p}_3]$	$[0\|M^{\rm p}_4]$	$[1\|M^{\rm p}_4]$	1	0
$[0\|M^{\rm s}_1]$	0.99707	0.00004	0.00015	0.00010	0.00208	0.00031	0.00025
$[0\|M^{\rm s}_2]$	0.00007	0.99727	0.00012	0.00004	0.00199	0.00028	0.00023
$[0\|M^{\rm s}_3]$	0.00004	0.00002	0.99845	0.00001	0.00117	0.00019	0.00012

Supplementary Table 4: Values of the weights used to define each secondary measurement procedure. Each of the three secondary outcome-0 measurement events, denoted $[0|M^{\rm s}_t]$ where $t \in \{1,2,3\}$ (the rows), is a probabilistic mixture of the four primary outcome-0 measurement events, denoted $[0|M^{\rm p}_{t'}]$ where $t' \in \{1,2,3,4\}$, and three processings thereof, denoted $[1|M^{\rm p}_4]$, 1, and 0 (the seven columns). The table presents the weights appearing in each such mixture. These are determined numerically by maximizing the function $C_{\rm M} = \frac{1}{3}\sum_{t=1}^{3} v^t_t$ (the average of the weights appearing in the shaded cells), which quantifies the closeness of the secondary procedures to the primary ones, subject to the constraint of operational equivalence between the uniform mixture of $M^{\rm s}_1$, $M^{\rm s}_2$ and $M^{\rm s}_3$ and a fair coin flip. The values presented are averages over 100 runs.

SUPPLEMENTARY NOTES

Supplementary Note 1: Elaboration of the notion of noncontextuality and the idealizations of previous proposals for tests

The notion of noncontextuality

In this article, we have used the operational notion of noncontextuality proposed in Supplementary Reference 1. According to this notion, one can distinguish noncontextuality for measurements and noncontextuality for preparations. To provide formal definitions, we must first review the notion of operational equivalence.

Recall that an operational theory specifies a set of physically possible measurements, $\mathcal{M}$, and a set of physically possible preparations, $\mathcal{P}$. Each measurement $M \in \mathcal{M}$ and preparation $P \in \mathcal{P}$ is assumed to be given as a list of instructions of what to do in the laboratory. An operational theory also specifies a function p, which determines, for every preparation $P \in \mathcal{P}$ and every measurement $M \in \mathcal{M}$, the probability distribution over the outcome X of the measurement when it is implemented on that preparation, $p(X|M, P)$.

Two measurement procedures, M and M', are said to be operationally equivalent if they have the same distribution over outcomes for all preparation procedures,

$$p(X|M, P) = p(X|M', P),\ \forall P \in \mathcal{P} \tag{1}$$

Two preparation procedures, P and P', are said to be operationally equivalent if they yield the same distribution over outcomes for all measurement procedures,

$$p(X|M, P') = p(X|M, P),\ \forall M \in \mathcal{M} \tag{2}$$

Any parameters that can be used to describe differences between the measurement procedures in a given operational equivalence class are considered to be part of the *measurement context*. Similarly, parameters that describe differences between preparation procedures in a given operational equivalence class are considered to be part of the *preparation context*. This terminological convention explains the suitability of the term *context-independent* or *noncontextual* for an ontological model wherein the representation of a given preparation or measurement depends *only* on the equivalence class to which it belongs (as defined below).

A *tomographically complete set* of preparation procedures, $\mathcal{P}_{\text{tomo}} \subseteq \mathcal{P}$, is defined as one that is sufficient for determining the statistics for any other preparation procedure, and hence is sufficient for deciding operational equivalence of measurements. In other words, one can equally well define operational equivalence of measurements M and M' by

$$p(X|M, P) = p(X|M', P),\ \forall P \in \mathcal{P}_{\text{tomo}} \tag{3}$$

Similarly, a tomographically complete set of measurement procedures, $\mathcal{M}_{\text{tomo}} \subseteq \mathcal{M}$, is defined as one that is sufficient for determining the statistics for any other measurement procedure, and hence is sufficient for deciding operational equivalence of preparations, such that we can define operational equivalence of preparations P and P' by

$$p(X|M, P') = p(X|M, P),\ \forall M \in \mathcal{M}_{\text{tomo}} \tag{4}$$

Note that if the tomographically complete set of preparations for a given system has infinite cardinality, then it is impossible to test operational equivalence experimentally. In quantum theory, the tomographically complete set for any finite-dimensional system has finite cardinality.

Recall that an ontological model of an operational theory specifies a space Λ of ontic states, where an ontic state gives the values of a set of classical variables that mediate the causal influence of the preparation on the measurement. An ontological model also specifies, for every preparation $P \in \mathcal{P}$, a distribution $\mu(\lambda|P)$. The idea is that when the preparation P is implemented on a system, it emerges from the preparation device in an ontic state λ, where λ need not be fixed by P but is instead obtained by sampling from the distribution $\mu(\lambda|P)$. Similarly, for every measurement $M \in \mathcal{M}$, an ontological model specifies the probabilistic response of the measurement to λ, specified as a conditional probability $\xi(X|M, \lambda)$ where X is a variable associated to the outcome of M. The idea here is that when an ontic state λ is fed into the measurement M, it need not fix the outcome X, but the outcome is sampled from the distribution $\xi(X|M, \lambda)$.

The assumption of *measurement noncontextuality* is that measurements that are operationally equivalent should be represented by the same conditional probability distributions in the ontological model,

$$\begin{aligned} & p(X|M, P) = p(X|M', P),\ \forall P \in \mathcal{P}_{\text{tomo}} \\ & \quad \rightarrow \xi(X|M, \lambda) = \xi(X|M', \lambda),\ \forall \lambda \in \Lambda. \end{aligned} \tag{5}$$

The assumption of *preparation noncontextuality* is that preparations that are operationally equivalent should be represented by the same distributions over ontic states in the ontological model

$$p(X|M,P) = p(X|M,P'),\ \forall M \in \mathcal{M}_{\text{tomo}}$$
$$\to \mu(\lambda|P) = \mu(\lambda|P'),\ \forall \lambda \in \Lambda. \tag{6}$$

A model is termed simply *noncontextual* if it is measurement noncontextual and preparation noncontextual.

We can summarize this as follows. The grounds for thinking that two measurement procedures are associated with the *same* observable, and hence that they are represented equivalently in the noncontextual model, is that they give equivalent statistics for all preparation procedures. Similarly, two preparations are represented equivalently in the noncontextual model only if they yield the same statistics for all measurements.

The notion of noncontextuality can be understood as a version of Leibniz's Principle of the Identity of Indiscernibles, specifically, the *physical* identity of *operational* indiscernibles. Other instances of the principle's use in physics include the inference from the lack of superluminal signals to the lack of superluminal causal influences (which justifies Bell's assumption of local causality [2]), and Einstein's inference from the operational indistinguishability of accelerating frames and frames fixed in a gravitational field to the physical equivalence of such frames. The question of whether nature admits of a noncontextual model can be understood as whether it adheres to this version of Leibniz's principle, at least within the framework of ontological models that underlies the discussion of noncontextuality.

It is argued in Supplementary Reference 1 that because the methodological principle underlying measurement noncontextuality is the same as the one underlying preparation noncontextuality, if one assumes the first, then one should also assume the second.

As is shown in Supplementary Reference 1, the traditional notion of noncontextuality, due to Kochen and Specker [3], can be understood as an application of measurement noncontextuality to projective measurements in quantum theory, but involves furthermore an additional assumption that projective measurements should have a *deterministic* response to the ontic state.

With these notions in hand, we turn to our criticisms of previous experiments designed to test the principle of noncontextuality.

Criticism of previous experiments seeking to test noncontextuality

Our first criticism of previous tests of noncontextuality is that the inequalities that they used (with the exception of Supplementary Reference 4) are only justified under the unwarranted idealization of noiseless measurements. We here expand on this criticism.

To implement an experimental test of noncontextuality, one requires a notion of noncontextuality that makes no reference to quantum theory, in particular, no reference to projective measurements. As such, it is necessary to first operationalize the traditional notion of noncontextuality (which refers to projective measurements) so that it can be applied without presuming the applicability of quantum theory to the experiment. The inequalities used in all previous experiments seeking to test noncontextuality have done so in a particular way. They have presumed that the experimentally-realized measurements are represented in the same manner as *projective* measurements are repesented according to the traditional notion of noncontextuality, namely, as responding *deterministically* to the ontic state. It is this assumption that is the source of the problem.

First, recall that determinism is not part of Bell's notion of local causality, such that for any no-go theorem based on local causality, one cannot avoid the contradiction by simply abandoning determinism. In other words, indeterminism does not provide a way out of Bell's theorem. The generalized notion of noncontextuality proposed in Supplementary Reference 1 improves upon the traditional notion by following Bell's lead and excising the notion of determinism from the notion of noncontextuality. For any no-go theorem based on the generalized notion, therefore, merely abandoning determinism is not sufficient to avoid contradiction.

Under this proposal, any appeal to the assumption of determinism must be *justified* from the principle of noncontextuality and from predictions of the operational theory. Such justifications are indeed possible, and play an important role in proofs of the failure of the generalized notion of noncontextuality (as we shall spell out in a moment). However, such justifications hold only under idealizations that are never realized in real experiments. An analogy with Bell tests highlights the problem.

Bell's 1964 argument [5] leveraged a prediction of quantum theory—that if the same measurement is implemented on two halves of a singlet state, then the outcomes will be perfectly anticorrelated—to *derive* facts about the assignment of measurement outcomes by the hidden variables, in particular that they must be deterministic. From these facts, Bell derived his 1964 inequality. But given that experimental correlations are never perfect, the assumptions on which this inequality is based are never satisfied in a real experiment. This is why experimentalists use other inequalities, such as the Clauser-Horne-Shimony-Holt inequality [6] or the Clauser-Horne inequality [7], to test local causality.

In Supplementary Reference 1, it was shown that if one makes an assumption of noncontextuality for preparations as well as for measurements, then one can *derive* the fact that projective measurements should respond deterministically to the ontic state. The inference relies on certain predictions of quantum theory, in particular, that for every projective rank-1 measurement, there is a basis of quantum states that makes its outcome perfectly predictable [1, 8]. However, perfect predictability only holds under the idealization of noiseless measurements, which is never achieved in practice. Therefore, one cannot use such considerations to justify the assumption that experimentally-realized measurements should have a deterministic response to the ontic state, unless one makes the unwarranted idealization that these measurements are noiseless.

Previous experiments for testing noncontextuality ought, therefore, to be considered as having the same status as would be granted to a putative experimental test of local causality using Bell's original 1964 inequality: neither can achieve its goal except under the unwarranted idealization of noiseless measurements.

More importantly, in Supplementary Reference 8, it was shown (by several different arguments) that noisy measurements *must* be modelled by indeterministic responses to the ontic state. One argument in favour of this conclusion is that to assume otherwise trivializes the notion of noncontextuality. For instance, if one assumes, as part of one's notion of noncontextuality, that noisy measurements have deterministic responses to the ontic state, then it becomes possible to demonstrate a failure of this notion of noncontextuality by a trivial experiment involving a measurement that ignores the system completely and instead generates an outcome by flipping a fair coin. The reason is as follows. If the measurement outcome is determined by a fair coin flip, then it is independent of the input state, and hence for all states each outcome occurs with probability $\frac{1}{2}$, meaning that the two outcomes are operationally equivalent. Given that they are operationally equivalent, if they are to be modelled noncontextually they must be modelled as having the *same* response to the ontic state. (In quantum theory, the fair coin flip is represented by the POVM $\{\frac{1}{2}\mathbb{I}, \frac{1}{2}\mathbb{I}\}$ where $\mathbb{I}$ is the identity operator, the operational equivalence of the two outcomes corresponds to the fact that they are represented by the same effect, namely $\frac{1}{2}\mathbb{I}$, and the assumption of noncontextuality for POVMs is that if two measurement outcomes are represented by the same effect, then they are represented by the same response function in the model.) But if, for the two outcomes, the response to the ontic state is the same and is deterministic, then for every ontic state, either both outcomes must receive probability 0 or both outcomes must receive probability 1, and each of these options is a logical contradiction.

Whatever else one might require of a proposed definition of the notion of noncontextuality, at minimum one should require that it capture a distinction between classical and quantum theories. Because a notion of noncontextuality that incorporates the assumption of determinism for noisy measurements can be ruled out by a classical experiment—indeed a trivial experiment consisting of a fair coin flip—it does not meet this minimum requirement and therefore is not one that is worthy of serious study.

Our second criticism of previous tests concerns the impossibility of realizing two experimental procedures in such a way that they are *exactly* operationally equivalent. No two experimental procedures ever give *precisely* the same statistics. In formal terms, for any two measurements M and M' that one realizes in the laboratory, it is never the case that one achieves precise equality in Supplementary Equation (3). Similarly, for any two preparations P and P' that one realizes in the laboratory, it is never the case that one achieves precisely equality in Supplementary Equation (4). In both cases, this is due to the fact that, in practice, one never quite achieves the experimental procedure that one intends to implement. The problem for an experimental test of noncontextuality, therefore, is that the conditions for applicability of the assumption of noncontextuality (the antecedents in the inferences of Supplementary Equations (5) and (6)) are, strictly speaking, never satisfied.

All previous experiments that have sought to test noncontextuality have used inequalities that are only justified under the unwarranted idealization that certain pairs of experimental operations are *exactly* operational equivalent. Even the experiment of Supplementary Reference 4, which unlike all other such tests did *not* make the unwarranted idealization of noiseless measurements, nonetheless made this second sort of unwarranted idealization. More precisely, the experiment of Supplementary Reference 4 tested an inequality whose derivation presumed a certain pair of preparation procedures to be represented equivalently in the ontological model even though the procedures were known to be merely *close* to operationally equivalent, rather than exactly so. Because such an inference does not, strictly speaking, follow from the principle of noncontextuality, the inequality that was violated in the experiment relied on an unwarranted idealization.

Supplementary Note 2: Derivation and tightness of the bound in our noncontextuality inequality

Derivation of bound

In the main text, we only provided an argument for why our two applications of the assumption of noncontextuality, Equations (3) and (5), implied that the quantity A must be bounded away from 1. Here we show that the explicit

value of this bound is $\frac{5}{6}$.

By definition,

$$A \equiv \frac{1}{6} \sum_{t\in\{1,2,3\}} \sum_{b\in\{0,1\}} p(X=b|M_t, P_{t,b}). \tag{7}$$

Substituting for $p(X{=}b|M_t, P_{t,b})$ the expression in terms of the distribution $\mu(\lambda|P_{t,b})$ and the response function $\xi(X=b|M_t,\lambda)$ given in Equation (1) in the main text, we have

$$A = \frac{1}{6} \sum_{t\in\{1,2,3\}} \sum_{b\in\{0,1\}} \sum_{\lambda\in\Lambda} \xi(X=b|M_t,\lambda)\mu(\lambda|P_{t,b}). \tag{8}$$

We now simply note that there is an upper bound on each response function that is independent of the value of b, namely,

$$\xi(X=b|M_t,\lambda) \leq \eta(M_t,\lambda), \tag{9}$$

where

$$\eta(M_t,\lambda) \equiv \max_{b'\in\{0,1\}} \xi(X=b'|M_t,\lambda). \tag{10}$$

We therefore have

$$A \leq \frac{1}{3} \sum_{t\in\{1,2,3\}} \sum_{\lambda\in\Lambda} \eta(M_t,\lambda) \left(\frac{1}{2} \sum_{b\in\{0,1\}} \mu(\lambda|P_{t,b}) \right), \tag{11}$$

Recalling that P_t is an equal mixture of $P_{t,0}$ and $P_{t,1}$, so that

$$\mu(\lambda|P_t) = \frac{1}{2}\mu(\lambda|P_{t,0}) + \frac{1}{2}\mu(\lambda|P_{t,1}), \tag{12}$$

we can rewrite the bound as simply

$$A \leq \frac{1}{3} \sum_{t\in\{1,2,3\}} \sum_{\lambda\in\Lambda} \eta(M_t,\lambda)\mu(\lambda|P_t). \tag{13}$$

But recalling Equation (5) from the main text,

$$\forall\lambda \in \Lambda : \mu(\lambda|P_1) = \mu(\lambda|P_2) = \mu(\lambda|P_3), \tag{14}$$

we see that the distribution $\mu(\lambda|P_t)$ is independent of t, so we denote it by $\nu(\lambda)$ and rewrite the bound as

$$A \leq \sum_{\lambda\in\Lambda} \left(\frac{1}{3} \sum_{t\in\{1,2,3\}} \eta(M_t,\lambda) \right) \nu(\lambda). \tag{15}$$

This last step is the first use of noncontextuality in the proof because Supplementary Equation (14) is derived from preparation noncontextuality and the operational equivalence of Equation (4) from the main text. It then follows that

$$A \leq \max_{\lambda\in\Lambda} \left(\frac{1}{3} \sum_{t\in\{1,2,3\}} \eta(M_t,\lambda) \right). \tag{16}$$

Therefore, if we can provide a nontrivial upper bound on $\frac{1}{3}\sum_t \eta(M_t,\lambda)$ for an arbitrary ontic state λ, we obtain a nontrivial upper bound on A. We infer constraints on the possibilities for the triple $(\eta(M_1,\lambda), \eta(M_2,\lambda), \eta(M_3,\lambda))$ from constraints on the possibilities for the triple $(\xi(X{=}0|M_1,\lambda), \xi(X{=}0|M_2,\lambda), \xi(X{=}0|M_3,\lambda))$.

The latter triple is constrained by Equation (7) from the main text, which in the case of $X=0$ reads

$$\frac{1}{3} \sum_{t\in\{1,2,3\}} \xi(X{=}0|M_t,\lambda) = \frac{1}{2}. \tag{17}$$

This is the second use of noncontextuality in our proof, because Supplementary Equation (17) is derived from the operational equivalence of Equation (2) from the main text and the assumption of measurement noncontextuality.

The fact that the range of each response function is $[0,1]$ implies that the vector $(\xi(X{=}0|M_1,\lambda), \xi(X{=}0|M_2,\lambda), \xi(X{=}0|M_3,\lambda))$ is constrained to the unit cube. The linear constraint of Supplementary Equation (17) implies that these vectors are confined to a two-dimensional plane. The intersection of the plane and the cube defines the polygon depicted in Supplementary Figure 1. The six vertices of this polygon have coordinates that are a permutation of $(1, \frac{1}{2}, 0)$. For every λ, the vector $(\xi(X{=}0|M_1,\lambda), \xi(X{=}0|M_2,\lambda), \xi(X{=}0|M_3,\lambda))$ corresponds to a point in the convex hull of these extreme points and given that $\frac{1}{3}\sum_t \eta(M_t,\lambda)$ is a convex function of this vector, it suffices to find a bound on the value of this function at the extreme points. If λ is the extreme point $(1, \frac{1}{2}, 0)$, then we have $(\eta(M_1,\lambda), \eta(M_2,\lambda), \eta(M_3,\lambda)) = (1, \frac{1}{2}, 1)$, and the other extreme points are simply permutations thereof. It follows that

$$\frac{1}{3}\sum_t \eta(M_t,\lambda) \leq \frac{5}{6}. \tag{18}$$

Substituting this bound into Supplementary Equation (16), we have our result.

Tightness of bound: two ontological models

In this section, we provide an explicit example of a noncontextual ontological model that saturates our noncontextuality inequality, thus proving that the noncontextuality inequality is tight, i.e., the upper bound of the inequality cannot be reduced any further for a noncontextual model.

We also provide an example of an ontological model that is preparation noncontextual but fails to be measurement noncontextual (i.e. it is measurement *contextual*) and that exceeds the bound of our noncontextuality inequality. This makes it clear that preparation noncontextuality alone does not suffice to justify the precise bound in our inequality, the assumption of measurement noncontextuality is a necessary ingredient as well. Given that we do not believe preparation noncontextuality on its own to be a reasonable assumption (as discussed in Supplementary Note 1), we highlight this fact only as a clarification of which features of the experiment are relevant for the particular bound that we obtain.

Note that there is no point inquiring about the bound for models that are measurement noncontextual but preparation contextual because, as shown in Supplementary Reference 1, quantum theory admits of models of this type—the ontological model wherein the pure quantum states are the ontic states (the ψ-complete ontological model in the terminology of Supplementary Reference 9) is of this sort.

For the two ontological models we present, we begin by specifying the ontic state space Λ. These are depicted in Supplementary Figures 2 and 3 as pie charts with each slice corresponding to a different element of Λ. We specify the six preparations $P_{t,b}$ by the distributions over Λ that they correspond to, denoted $\mu(\lambda|P_{t,b})$ (middle left of Supplementary Figures 2 and 3). We specify the three measurements M_t by the response functions for the $X=0$ outcome, denoted $\xi(0|M_t,\lambda)$ (top right of Supplementary Figures 2 and 3). Finally, we compute the operational probabilities for the various preparation-measurement pairs, using Equation (1) from the main text, and display the results in the 6×4 upper-left-hand corner of Supplementary Tables 1 and 2.

In the remainder of each table, we display the operational probabilities for the effective preparations, P_t, which are computed from the operational probabilities for the $P_{t,b}$ and the fact that P_t is the uniform mixture of $P_{t,0}$ and $P_{t,1}$. We also display the operational probabilities for the effective measurement M_*, which is computed from the operational probabilities for the M_t and the fact that M_* is a uniform mixture of M_1, M_2 and M_3.

From the tables, we can verify that our two ontological models imply the operational equivalences that we use in the derivation of our noncontextuality inequality. Specifically, the three preparations P_1, P_2 and P_3 yield exactly the same statistics for all of the measurements, and the measurement M_* is indistinguishable from a fair coin flip for all the preparations.

Supplementary Figures 2 and 3 also depict $\mu(\lambda|P_t)$ for $t \in \{1,2,3\}$ for each model (bottom left). These are determined from the $\mu(\lambda|P_{t,b})$ via Supplementary Equation (12). Similarly, the response function $\xi(0|M_*,\lambda)$, which is determined from $\xi(X=b|M_*,\lambda) = \frac{1}{3}\sum_{t\in\{1,2,3\}} \xi(X=b|M_t,\lambda)$, is displayed in each case (bottom right).

Given the operational equivalence of P_1, P_2 and P_3, an ontological model is preparation noncontextual if and only if $\mu(\lambda|P_1) = \mu(\lambda|P_2) = \mu(\lambda|P_3)$ for all $\lambda \in \Lambda$. We see, therefore, that both models are preparation noncontextual.

Similarly given the operational equivalence of M_* and a fair coin flip, an ontological model is measurement noncontextual if and only if $\xi(0|M_*,\lambda) = \frac{1}{2}$ for all $\lambda \in \Lambda$. We see, therefore, that only the first model is measurement noncontextual.

Note that in the second model, M_* manages to be operationally equivalent to a fair coin flip, despite the fact that when one conditions on a given ontic state λ, it does not have a uniformly random response. This is possible only because the set of distributions is restricted in scope, and the overlaps of these distributions with the response functions always generates the uniformly random outcome. This highlights how an ontological model can do justice to the operational probabilities while failing to be noncontextual.

Finally, using the operational probabilities in the tables, one can compute the value of A for each model. It is determined entirely by the operational probabilities in the shaded cells. One thereby confirms that $A = \frac{5}{6}$ in the first model, while $A = \frac{9}{10}$ in the second model.

Supplementary Note 3: Constructing the secondary procedures from the primary ones

Secondary preparations in quantum theory

As noted in the main text, it is easiest to describe the details of our procedure for defining secondary preparations if we make the assumption that quantum theory correctly describes the experiment. Further on, we will describe the procedure for a generalised probabilistic theory (GPT).

Figure 1 in the main text described how to define the secondary preparations if the primary preparations deviate from the ideal only *within* the $\boldsymbol{x}-\boldsymbol{z}$ plane of the Bloch sphere. Here, we consider the case where the six primary preparations deviate from the ideals within the bulk of the Bloch sphere. The fact that our proof only requires that the secondary preparations satisfy Equation (10) from the main text means that the different pairs, $P^{\rm s}_{t,0}$ and $P^{\rm s}_{t,1}$ for $t \in \{1,2,3\}$, need not all mix to the center of the Bloch sphere, but only to the *same* state. It follows that the three pairs need not be coplanar in the Bloch sphere. Note, however, for any *two* values, t and t', the four preparations $P^{\rm s}_{t,0}, P^{\rm s}_{t,1}, P^{\rm s}_{t',0}, P^{\rm s}_{t',1}$ do need to be coplanar.

Any mixing procedure defines a map from each of the primary preparations $P^{\rm p}_{t,b}$ to the corresponding secondary preparation $P^{\rm s}_{t,b}$, which can be visualized as a motion of the corresponding point within the Bloch sphere. To ensure that the six secondary preparations approximate well the ideal preparations while also defining mixed preparations $P^{\rm s}_1$, $P^{\rm s}_2$ and $P^{\rm s}_3$ that satisfy the appropriate operational equivalences, the mixing procedure must allow for motion in the $\pm\boldsymbol{y}$ direction. Consider what happens if one tries to achieve such motion *without* supplementing the primary set with the eigenstates of $\boldsymbol{\sigma}\cdot\boldsymbol{y}$. A given point that is biased towards $-\boldsymbol{y}$ can be moved in the $+\boldsymbol{y}$ direction by mixing it with another point that has less bias in the $-\boldsymbol{y}$ direction. However, because the primary preparations are widely separated within the $\boldsymbol{x}-\boldsymbol{z}$ plane, achieving a small motion in $+\boldsymbol{y}$ direction in this fashion comes at the price of a large motion within the $\boldsymbol{x}-\boldsymbol{z}$ plane, implying a significant motion away from the ideal. This problem is particularly pronounced if the primary points are very close to coplanar.

The best way to move a given point in the $\pm\boldsymbol{y}$ direction is to mix it with a point that is at roughly the same location within the $\boldsymbol{x}-\boldsymbol{z}$ plane, but displaced in the $\pm\boldsymbol{y}$ direction. This scheme, however, would require supplementing the primary set with one or two additional preparations for every one of its elements. Supplementing the original set with just the two eigenstates of $\boldsymbol{\sigma}\cdot\boldsymbol{y}$ constitutes a good compromise between keeping the number of preparations low and ensuring that the secondary preparations are close to the ideal. Because the $\boldsymbol{\sigma}\cdot\boldsymbol{y}$ eigenstates have the greatest possible distance from the $\boldsymbol{x}-\boldsymbol{z}$ plane, they can be used to move any point close to that plane in the $\pm\boldsymbol{y}$ direction while generating only a modest motion within the $\boldsymbol{x}-\boldsymbol{z}$ plane.

Secondary measurements in quantum theory

Just as with the case of preparations, we solve the problem of no strict statistical equivalences for measurements by noting that from the primary set of measurements, $M^{\rm p}_1$, $M^{\rm p}_2$ and $M^{\rm p}_3$, one can infer the statistics of a large family of measurements, and one can find three measurements within this family, called the secondary measurements and denoted $M^{\rm s}_1$, $M^{\rm s}_2$ and $M^{\rm s}_3$, such that their mixture, $M^{\rm s}_*$, satisfies the operational equivalence of Equation (2) in the main text *exactly*. To give the details of our approach, it is again useful to begin with the quantum description.

A geometric visualization of the construction is also possible in this case. Just as a density operator can be written $\rho = \frac{1}{2}(\mathbb{I} + \boldsymbol{r}\cdot\boldsymbol{\sigma})$ to define a three-dimensional Bloch vector $\boldsymbol{r}$, an effect can be written $E = \frac{1}{2}(e_0\mathbb{I} + \boldsymbol{e}\cdot\boldsymbol{\sigma})$ to define a four-dimensional Bloch-like vector $(e_0, \boldsymbol{e})$, whose four components we will call the $\mathbb{I}$, $\boldsymbol{x}$, $\boldsymbol{y}$ and $\boldsymbol{z}$ components. Note that $e_0 = \mathrm{tr}(E)$, while $e_x = \mathrm{tr}(\boldsymbol{\sigma}\cdot\boldsymbol{x}E)$ and so forth. The eigenvalues of E are expressed in terms of these components as $\frac{1}{2}(e_o \pm |\boldsymbol{e}|)$. Consequently, the constraint that $0 \leq E \leq \mathbb{I}$ takes the form of three inequalities $0 \leq e_o \leq 2$, $|\boldsymbol{e}| \leq e_0$ and $|\boldsymbol{e}| \leq 2 - e_0$. This corresponds to the intersection of two cones. For the case $e_y = 0$, the Bloch representation of the effect space is three-dimensional and is displayed in Supplementary Figure 4. When portraying binary-outcome

measurements associated to a POVM $\{E, \mathbb{I} - E\}$ in this representation, it is sufficient to portray the Bloch-like vector $(e_0, \boldsymbol{e})$ for outcome E alone, given that the vector for $\mathbb{I} - E$ is simply $(2 - e_0, -\boldsymbol{e})$. Similarly, to describe any mixture of two such POVMs, it is sufficient to describe the mixture of the effects corresponding to the first outcome.

The family of measurements that is defined in terms of the primary set is slightly different than what we had for preparations. The reason is that each primary measurement on its own generates a family of measurements by probabilistic post-processing of its outcome. If we denote the outcome of the original measurement by X and that of the processed measurement by X', then the probabilistic processing is a conditional probability $p(X'|X)$. It is sufficient to determine the convexly-extremal post-processings, since all others can be obtained from these by mixing. For the case of binary outcome measurements considered here, there are just four extremal post-processings: the identity process, $p(X'|X) = \delta_{X',X}$; the process that flips the outcome, $p(X'|X) = \delta_{X',X\oplus 1}$; the process that always generates the outcome $X' = 0$, $p(X'|X) = \delta_{X',0}$; and the process that always generates the outcome $X' = 1$, $p(X'|X) = \delta_{X',1}$. Applying these to our three primary measurements, we obtain eight measurements in all: the two that generate a fixed outcome, the three originals, and the three originals with the outcome flipped. If the set of primary measurements corresponded to the ideal set, then the eight extremal post-processings would correspond to the observables $0, \mathbb{I}, \boldsymbol{\sigma}\cdot\boldsymbol{n_1}, -\boldsymbol{\sigma}\cdot\boldsymbol{n_1}, \boldsymbol{\sigma}\cdot\boldsymbol{n_2}, -\boldsymbol{\sigma}\cdot\boldsymbol{n_2}, \boldsymbol{\sigma}\cdot\boldsymbol{n_3}, -\boldsymbol{\sigma}\cdot\boldsymbol{n_3}$. In practice, the last six measurements will be unsharp. These eight measurements can then be mixed probabilistically to define the family of measurements from which the secondary measurements must be chosen. We refer to this family as the *convex hull of the post-processings* of the primary set.

We will again start with a simplified example, wherein the primary measurements have Bloch-like vectors with vanishing component along $\boldsymbol{y}$, $e_y = 0$, and unit component along $\mathbb{I}$, $e_0 = 1$, so that $E = \frac{1}{2}(\mathbb{I} + e_x\boldsymbol{\sigma}\cdot\boldsymbol{x} + e_z\boldsymbol{\sigma}\cdot\boldsymbol{z})$. In this case, the constraint $0 \leq E \leq \mathbb{I}$ reduces to $|\boldsymbol{e}| \leq 1$, which is the same constraint that applies to density operators confined to the $\boldsymbol{x} - \boldsymbol{z}$ plane of the Bloch sphere. Here, the only deviation from the ideal is within this plane, and the construction is precisely analogous to what is depicted in Figure 1 of the main text.

Unlike the case of preparations, however, the primary measurements can deviate from the ideal in the $\mathbb{I}$ direction, that is, E may have a component along $\mathbb{I}$ that deviates from 1, which corresponds to introducing a state-independent bias on the outcome of the measurement. This is where the extremal post-processings yielding the constant-outcome measurements corresponding to the observables 0 and $\mathbb{I}$ come in. They allow one to move in the $\pm\mathbb{I}$ direction.

Supplementary Figure 4 presents an example wherein the primary measurements have Bloch-like vectors that deviate from the ideal not only within the $\boldsymbol{x} - \boldsymbol{z}$ plane, but in the $\mathbb{I}$ direction as well (it is still presumed, however, that all components in the $\boldsymbol{y}$ direction are vanishing).

In practice, of course, the $\boldsymbol{y}$ component of our measurements never vanishes precisely either. We therefore apply the same trick as we did for the preparations. We supplement the set of primary measurements with an additional measurement, denoted M_4^{p}, that ideally corresponds to the observable $\boldsymbol{\sigma}\cdot\boldsymbol{y}$. The post-processing which flips the outcome then corresponds to the observable $-\boldsymbol{\sigma}\cdot\boldsymbol{y}$. Mixing the primary measurements with M_4^{p} and its outcome-flipped counterpart allows motion in the $\pm\boldsymbol{y}$ direction within the Bloch cone.

Note that the capacity to move in both the $+\boldsymbol{y}$ and the $-\boldsymbol{y}$ direction is critical for achieving the operational equivalence of Equation (2) in the main text, because if the secondary measurements had a common bias in the $\boldsymbol{y}$ direction, they could not mix to the POVM $\{\mathbb{I}/2, \mathbb{I}/2\}$ as Equation (9) from the main text requires. For the preparations, by contrast, supplementing the primary set by just *one* of the eigenstates of $\boldsymbol{\sigma}\cdot\boldsymbol{y}$ would still work, given that the mixed preparations P_t^{s} do not need to coincide with the completely mixed state $\mathbb{I}/2$.

The secondary measurements M_1^{s}, M_2^{s} and M_3^{s} are then chosen from the convex hull of the post-processings of the $M_1^{\mathrm{p}}, M_2^{\mathrm{p}}, M_3^{\mathrm{p}}, M_4^{\mathrm{p}}$. Without this supplementation, it may be impossible to find secondary measurements that define an M_*^{s} that satisfies the operational equivalences while providing a good approximation to the ideal measurements.

In all, under the extremal post-processings of the supplemented set of primary measurements, we obtain ten points which ideally correspond to the observables $0, \mathbb{I}, \boldsymbol{\sigma}\cdot\boldsymbol{n_1}, -\boldsymbol{\sigma}\cdot\boldsymbol{n_1}, \boldsymbol{\sigma}\cdot\boldsymbol{n_2}, -\boldsymbol{\sigma}\cdot\boldsymbol{n_2}, \boldsymbol{\sigma}\cdot\boldsymbol{n_3}, -\boldsymbol{\sigma}\cdot\boldsymbol{n_3}, \boldsymbol{\sigma}\cdot\boldsymbol{y}$, and $-\boldsymbol{\sigma}\cdot\boldsymbol{y}$.

Note that the outcome-flipped versions of the three primary measurements are not critical for defining a good set of secondary measurements, and indeed we find that we can dispense with them and still obtain good results. This is illustrated in the example of Supplementary Figure 4.

Secondary preparations and measurements in generalised probabilistic theories

We do not want to presuppose that our experiment is well fit by a quantum description. Therefore instead of working with density operators and POVMs, we work with GPT states and effects, which are inferred from the

matrix D^{p}

$$D^{\mathrm{p}} = \begin{pmatrix} p^1_{1,0} & p^1_{1,1} & \cdots & p^1_{4,0} & p^1_{4,1} \\ p^2_{1,0} & p^2_{1,1} & \cdots & p^2_{4,0} & p^2_{4,1} \\ p^3_{1,0} & p^3_{1,1} & \cdots & p^3_{4,0} & p^3_{4,1} \\ p^4_{1,0} & p^4_{1,1} & \cdots & p^4_{4,0} & p^4_{4,1} \end{pmatrix}. \tag{19}$$

where

$$p^{t'}_{t,b} \equiv p(0|M^{\mathrm{p}}_{t'}, P^{\mathrm{p}}_{t,b}) \tag{20}$$

is the probability of obtaining outcome 0 in the t'th measurement that was actually realized in the experiment (recall that we term this measurement primary and denote it by $M^{\mathrm{p}}_{t'}$), when it follows the (t,b)th preparation that was actually realized in the experiment (recall that we term this preparation primary and denote it by $P^{\mathrm{p}}_{t,b}$). These probabilities are estimated by fitting the raw experimental data (which are merely finite samples of the true probabilities) to a GPT; we postpone the description of this procedure to Supplementary Note 4.

The rows of the D^{p} matrix define the GPT effects. We denote the vector defined by the tth row, which is associated to the measurement event $[0|M^{\mathrm{p}}_t]$ (obtaining the 0 outcome in the primary measurement M^{p}_t), by $\mathbf{M}^{\mathrm{p}}_t$. Similarly, the columns of this matrix define the GPT states. We denote the vector associated to the (t,b)th column, which is associated to the primary preparation $P^{\mathrm{p}}_{t,b}$, by $\mathbf{P}^{\mathrm{p}}_{t,b}$.

As described in the main text, we define the *secondary* preparation $P^{\mathrm{s}}_{t,b}$ by a probabilistic mixture of the primary preparations. Thus, the GPT state of the secondary preparation is a vector $\mathbf{P}^{\mathrm{s}}_{t,b}$ that is a probabilistic mixture of the $\mathbf{P}^{\mathrm{p}}_{t,b}$,

$$\mathbf{P}^{\mathrm{s}}_{t,b} = \sum_{t'=1}^{4} \sum_{b'=0}^{1} u^{t,b}_{t',b'} \mathbf{P}^{\mathrm{p}}_{t',b'}, \tag{21}$$

where the $u^{t,b}_{t',b'}$ are the weights in the mixture.

A secondary measurement $M^{\mathrm{s}}_{t'}$ is obtained from the primary measurements in a similar fashion, but in addition to probabilistic mixtures, one must allow certain post-processings of the measurements, in analogy to the quantum case described above.

The set of all post-processings of the primary outcome-0 measurement events has extremal elements consisting of the outcome-0 measurement events themselves together with: the measurement event that *always* occurs, which is represented by the vector of probabilities where every entry is 1, denoted $\mathbf{1}$; the measurement event that *never* occurs (so that outcome 1 is certain instead), which is represented by the vector of probabilities where every entry is 0, denoted $\mathbf{0}$; and the outcome-flipped measurement events, which are represented by the vector $\mathbf{1} - \mathbf{M}^{\mathrm{p}}_t$.

We can therefore define our three secondary outcome-0 measurement events as probabilistic mixtures of the four primary ones as well as the extremal post-processings mentioned above, that is

$$\mathbf{M}^{\mathrm{s}}_t = \sum_{t'=1}^{4} v^t_{t'} \mathbf{M}^{\mathrm{p}}_{t'} + v^t_{\mathbf{0}} \mathbf{0} + v^t_{\mathbf{1}} \mathbf{1} + \sum_{t''=1}^{4} v^t_{\neg t''} (\mathbf{1} - \mathbf{M}^{\mathrm{p}}_{t''}), \tag{22}$$

where for each t, the vector of weights in the mixture is $(v^t_1, v^t_2, v^t_3, v^t_4, v^t_{\mathbf{0}}, v^t_{\mathbf{1}}, v^t_{\neg 1}, v^t_{\neg 2}, v^t_{\neg 3}, v^t_{\neg 4})$. We see that this is a particular type of linear transformation on the rows.

Again, as mentioned in the discussion of the quantum case, we can in fact limit the post-processing to exclude the outcome-flipped measurement events for M_1, M_2 and M_3, keeping only the outcome-flipped event for M_4, and still obtain good results. Thus we found it sufficient to search for secondary outcome-0 measurement events among those of the form

$$\mathbf{M}^{\mathrm{s}}_t = \sum_{t'=1}^{4} v^t_{t'} \mathbf{M}^{\mathrm{p}}_{t'} + v^t_{\mathbf{0}} \mathbf{0} + v^t_{\mathbf{1}} \mathbf{1} + v^t_{\neg 4} (\mathbf{1} - \mathbf{M}^{\mathrm{p}}_4), \tag{23}$$

where for each t, the vector of weights in the mixture is $(v^t_1, v^t_2, v^t_3, v^t_4, v^t_{\mathbf{0}}, v^t_{\mathbf{1}}, v^t_{\neg 4})$.

Returning to the preparations, we choose the weights $u^{t,b}_{t',b'}$ to maximize the function

$$C_{\mathrm{P}} \equiv \frac{1}{6} \sum_{t=1}^{3} \sum_{b=0}^{1} u^{t,b}_{t,b} \tag{24}$$

subject to the linear constraint

$$\frac{1}{2}\sum_b \mathbf{P}^{\mathrm{s}}_{1,b} = \frac{1}{2}\sum_b \mathbf{P}^{\mathrm{s}}_{2,b} = \frac{1}{2}\sum_b \mathbf{P}^{\mathrm{s}}_{3,b}, \tag{25}$$

as noted in the main text. This optimization ensures that the secondary preparations are as close as possible to the primary ones while ensuring that they satisfy the relevant operational equivalence *exactly.* Supplementary Table 3 reports the weights $u^{t,b}_{t',b'}$ that were obtained from this optimization procedure, averaged over the 100 runs of the experiment. As noted in the main text, these weights yield $C_{\mathrm{P}} = 0.9969 \pm 0.0001$, indicating that the secondary preparations are indeed very close to the primary ones.

The scheme for finding the weights $(v^t_1, v^t_2, v^t_3, v^t_4, v^t_{\mathbf{0}}, v^t_{\mathbf{1}}, v^t_{\neg 4})$ that define the secondary measurements is analogous. Using a linear program, we find the vector of such weights that maximizes the function

$$C_{\mathrm{M}} \equiv \frac{1}{3}\sum_{t=1}^{3} v^t_t, \tag{26}$$

subject to the constraint that

$$\mathbf{M}^{\mathrm{s}}_* = \frac{1}{2}\mathbf{1}, \tag{27}$$

where $\mathbf{M}^{\mathrm{s}}_* \equiv \frac{1}{3}\sum_{t=1}^{3}\mathbf{M}^{\mathrm{s}}_t$. A high value of C_{M} signals that each of the three secondary measurements is close to the corresponding primary one. Supplementary Table 4 reports the weights we obtain from this optimization procedure, averaged over the 100 runs of the experiment. These weights yield $C_{\mathrm{M}} = 0.9976 \pm 0.0001$, again indicating the closeness of the secondary measurements to the primary ones.

This optimization defines the precise linear transformation of the rows of D^{p} and the linear transformation of the columns of D^{p} that serve to define the secondary preparations and measurements. By combining the operations on the rows and on the columns, we obtain from D^{p} a 3×6 matrix, denoted D^{s}, whose entries $s^{t'}_{t,b}$ are

$$\sum_{\tau=1}^{4}\sum_{\beta=0}^{1} u^{t,b}_{\tau,\beta}\left[\sum_{\tau'=1}^{4} v^{t'}_{\tau'} p^{\tau'}_{\tau,\beta} + v^{t'}_{\mathbf{0}}0 + v^{t'}_{\mathbf{1}}1 + v^{t'}_{\neg 4}(1 - p^4_{\tau,\beta})\right] \tag{28}$$

where $t', t \in \{1,2,3\}$, $b \in \{0,1\}$. This matrix describes the secondary preparations $P^{\mathrm{s}}_{t,b}$ and measurements $M^{\mathrm{s}}_{t'}$. The component $s^{t'}_{t,b}$ of this matrix describes the probability of obtaining outcome 0 in measurement $M^{\mathrm{s}}_{t'}$ on preparation $P^{\mathrm{s}}_{t,b}$, that is,

$$s^{t'}_{t,b} \equiv p(0|M^{\mathrm{s}}_{t'}, P^{\mathrm{s}}_{t,b}). \tag{29}$$

These probabilities are the ones that are used to calculate the value of A via Equation (6) of the main text.

Supplementary Note 4: Data analysis

Fitting the raw data to a generalised probabilistic theory

In our experiment we perform four measurements on each of eight input states. If we define $r^{t'}_{t,b}$ as the fraction of '0' outcomes returned by measurement $M_{t'}$ on preparation $P_{t,b}$, the results can be summarized in a 4×8 matrix of raw data, D^{r}, defined as:

$$D^{\mathrm{r}} = \begin{pmatrix} r^1_{1,0} & r^1_{1,1} & \cdots & r^1_{4,0} & r^1_{4,1} \\ r^2_{1,0} & r^2_{1,1} & \cdots & r^2_{4,0} & r^2_{4,1} \\ r^3_{1,0} & r^3_{1,1} & \cdots & r^3_{4,0} & r^3_{4,1} \\ r^4_{1,0} & r^4_{1,1} & \cdots & r^4_{4,0} & r^4_{4,1} \end{pmatrix}. \tag{30}$$

Each row of D^{r} corresponds to a measurement, ordered from top to bottom as M_1, M_2, M_3, and M_4. Similary, the columns are labelled from left to right as $P_{1,0}$, $P_{1,1}$, $P_{2,0}$, $P_{2,1}$, $P_{3,0}$, $P_{3,1}$,$P_{4,0}$, and $P_{4,1}$.

In order to test the assumption that three independent binary-outcome measurements are tomographically complete for our system, we fit the raw data to a matrix, D^{p}, of primary data defined in Supplementary Equation (19). D^{p} contains the outcome probabilities of four measurements on eight states in the GPT-of-best-fit to the raw data. We fit to a GPT in which three 2-outcome measurements are tomographically complete, which we characterize with the following result.

Proposition 1 *A matrix* $D^{\rm p}$ *can arise from a GPT in which three two-outcome measurements are tomographically complete if and (with a measure zero set of exceptions) only if* $ap^1_{t,b}+bp^2_{t,b}+cp^3_{t,b}+dp^4_{t,b}-1=0$ *for some real constants* $\{a,b,c,d\}$.

Proof. We begin with the "only if" part. Following [10, 11], if a set of two-outcome measurements M_A, M_B, M_C (called *fiducial* measurements) are tomographically complete for a system, then the state of the system given a preparation P can be specified by the vector

$$\mathbf{p} = \begin{pmatrix} 1 \\ p(0|M_A,P) \\ p(0|M_B,P) \\ p(0|M_C,P) \end{pmatrix} \tag{31}$$

(where the first entry indicates that the state is normalized). In [10, 11] it is shown that convexity then requires that the probability of outcome '0' for any measurement M is given by $\mathbf{r}\cdot\mathbf{p}$ for some vector $\mathbf{r}$. Let $\mathbf{r}_1,\mathbf{r}_2,\mathbf{r}_3,\mathbf{r}_4$ correspond to outcome '0' of the measurements M_1, M_2, M_3, M_4, and note that the measurement event that *always* occurs, regardless of the preparation (e.g. the event of obtaining either outcome '0' or '1' in any binary-outcome measurement), must be represented by $\mathbf{r}_{\mathbb{I}} = (1,0,0,0)$. Since the $\mathbf{r}_1,\mathbf{r}_2,\mathbf{r}_3,\mathbf{r}_4,\mathbf{r}_{\mathbb{I}}$ are a set of five four-dimensional vectors, they must be linearly dependent:

$$a'\mathbf{r}_1 + b'\mathbf{r}_2 + c'\mathbf{r}_3 + d'\mathbf{r}_4 + e'\mathbf{r}_{\mathbb{I}} = 0 \tag{32}$$

with $(a',b',c',d',e') \neq (0,0,0,0,0)$. The set of $\mathbf{r}$ for which e' *must* be zero are those where $\mathbf{r}_{\mathbb{I}}$ is not in the span of $\mathbf{r}_1,\mathbf{r}_2,\mathbf{r}_3,\mathbf{r}_4$, which is a set of measure zero. Hence we can generically ensure $e' \neq 0$ and divide Supplementary Equation (32) through by $-e'$ to obtain

$$a\mathbf{r}_1 + b\mathbf{r}_2 + c\mathbf{r}_3 + d\mathbf{r}_4 - \mathbf{r}_{\mathbb{I}} = 0 \tag{33}$$

where $a = -a'/e'$, $b = -b'/e'$ and so on.

Finally, letting $\mathbf{p}_{t,b}$ denote the column vector of the form of Supplementary Equation (31) that is associated to the preparation $P_{t,b}$, and noting that by definition

$$p^{t'}_{t,b} = \mathbf{r}_{t'}\cdot\mathbf{p}_{t,b}, \tag{34}$$

we see that by taking the dot product of Supplementary Equation (33) with each $\mathbf{p}_{t,b}$, we obtain the desired constraint on $D_{\rm p}$.

For the "if" part, we assume the constraint and demonstrate that there exists a triple of binary-outcome measurements, M_A, M_B, and M_C, that are tomographically complete for the GPT. To establish this, it is sufficient to take the fiducial set, M_A, M_B and M_C, to be M_1, M_2, and M_3, so that preparation $P_{t,b}$ corresponds to the vector

$$\mathbf{p}_{t,b} = \begin{pmatrix} 1 \\ p^1_{t,b} \\ p^2_{t,b} \\ p^3_{t,b} \end{pmatrix}. \tag{35}$$

In this case, we can recover $D^{\rm p}$ if M_1, M_2, and M_3 are represented by $\mathbf{r}_1 = (0,1,0,0)$, $\mathbf{r}_2 = (0,0,1,0)$ and $\mathbf{r}_3 = (0,0,0,1)$, whilst the assumed constraint implies that $\mathbf{r}_4 = -(-1,a,b,c)/d$. ■

Geometrically, the proposition dictates that the eight columns of $D^{\rm p}$ lie on the 3-dimensional hyperplane defined by the constants $\{a,b,c,d\}$.

To find the GPT-of-best-fit we fit a 3-d hyperplane to the eight 4-dimensional points that make up the columns of $D^{\rm r}$. We then map each column of $D^{\rm r}$ to its closest point on the hyperplane, and these eight points will make up the columns of $D^{\rm p}$. We use a weighted total least-squares procedure [12, 13] to perform this fit. Each element of $D^{\rm r}$ has an uncertainty, $\Delta r^{t'}_{t,b}$, which is estimated assuming the dominant source of error is the statistical error arising from Poissonian counting statistics. We define the *weighted distance*, $\chi_{t,b}$, between the (t,b) column of $D^{\rm r}$ and $D^{\rm p}$ as $\chi_{t,b} = \sqrt{\sum_{t'=1}^{4}\left(r^{t'}_{t,b}-p^{t'}_{t,b}\right)^2 / \left(\Delta r^{t'}_{t,b}\right)^2}$. Finding the best-fitting hyperplane can be summarized as the following minimization problem:

$$\begin{aligned} \underset{\{p^i_{t,b},a,b,c,d\}}{\text{minimize}} \quad & \chi^2 = \sum_{t=1}^{4}\sum_{b=0}^{1}\chi^2_{t,b}, \\ \text{subject to} \quad & ap^1_{t,b}+bp^2_{t,b}+cp^3_{t,b}+dp^4_{t,b}-1=0 \\ & \forall\, t=1,\dots,4,\ b=0,1. \end{aligned} \tag{36}$$

The optimization problem as currently phrased is a problem in 36 variables—the 32 elements of D^{p} together with the hyperplane parameters $\{a,b,c,d\}$. We can simplify this by first solving the simpler problem of finding the weighted distance $\chi_{t,b}$ between the (t,b) column of D^{r} and the hyperplane $\{a,b,c,d\}$. This can be phrased as the following 8-variable optimization problem:

$$\begin{aligned} \underset{\{p^1_{t,b},p^2_{t,b},p^3_{t,b},p^4_{t,b}\}}{\text{minimize}} \quad & \chi^2_{t,b} = \sum_{t'=1}^{4} \frac{(r^{t'}_{t,b}-p^{t'}_{t,b})^2}{\left(\Delta r^{t'}_{t,b}\right)^2}, \\ \text{subject to} \quad & ap^1_{t,b}+bp^2_{t,b}+cp^3_{t,b}+dp^4_{t,b}-1=0. \end{aligned} \tag{37}$$

Using the method of Lagrange multipliers [12], we define the Lagrange function $\Gamma = \chi^2_{t,b} + \gamma(ap^1_{t,b}+bp^2_{t,b}+cp^3_{t,b}+dp^4_{t,b}-1)$, where γ denotes the Lagrange multiplier, then simultaneously solve

$$\frac{\partial\Gamma}{\partial\gamma} = 0 \tag{38}$$

and

$$\frac{\partial\Gamma}{\partial p^{t'}_{t,b}} = 0,\ t' = 1,\ldots,4 \tag{39}$$

for the variables γ, $p^1_{t,b}$, $p^2_{t,b}$, $p^3_{t,b}$, and $p^4_{t,b}$. Substituting the solutions for $p^1_{t,b}$, $p^2_{t,b}$, $p^3_{t,b}$ and $p^4_{t,b}$ into Supplementary Equation (37) we find

$$\chi^2_{t,b} = \frac{(ar^1_{t,b}+br^2_{t,b}+cr^3_{t,b}+dr^4_{t,b}-1)^2}{\left(a\Delta r^1_{t,b}\right)^2+\left(b\Delta r^2_{t,b}\right)^2+\left(c\Delta r^3_{t,b}\right)^2+\left(d\Delta r^4_{t,b}\right)^2}, \tag{40}$$

which now only contains the variables a, b, c, and d.

The hyperplane-finding problem can now be stated as the following four-variable optimization problem:

$$\underset{\{a,b,c,d\}}{\text{minimize}} \quad \chi^2 = \sum_{t=1}^{4}\sum_{b=0}^{1}\chi^2_{t,b} \tag{41}$$

which we solve numerically.

The χ^2 parameter returned by the fitting procedure is a measure of the goodness-of-fit of the hyperplane to the data. Since we are fitting eight data points to a hyperplane defined by four fitting parameters $\{a,b,c,d\}$, we expect the χ^2 parameter to be drawn from a χ^2 distribution with four degrees of freedom [13], which has a mean of 4. As stated in the main text, we ran our experiment 100 times and obtained 100 independent χ^2 parameters; these have a mean of 3.9 ± 0.3. In addition we performed a more stringent test of the fit of the model to the data by summing the counts from all 100 experimental runs before performing a single fit. This fit returns a χ^2 of 4.33, which has a p-value of 36%. The outcomes of these tests are consistent with our assumption that the raw data can be explained by a GPT in which three 2-outcome measurements are tomographically complete and which also exhibits Poissonian counting statistics. Had the fitting procedure returned χ^2 values that were much higher, this would have indicated that the theoretical description of the preparation and measurement procedures required more than three degrees of freedom. On the other hand, had the fitting returned an average χ^2 much lower than 4, this would have indicated that we had overestimated the amount of uncertainty in our data.

After finding the hyperplane-of-best-fit $\{a,b,c,d\}$, we find the points on the hyperplane that are closest to each column of D^{r}. This is done by numerically solving for $p^1_{t,b}$, $p^2_{t,b}$, $p^3_{t,b}$, and $p^4_{t,b}$ in (37) for each value of (t,b). The point on the hyperplane closest to the (t,b) column of D^{r} becomes the (t,b) column of D^{p}. The matrix D^{p} is then used to find the secondary preparations and measurements.

Why is fitting to a GPT necessary?

It is clear that one needs to assume that the measurements one has performed form a tomographically complete set, otherwise statistical equivalence relative to those measurements does not imply statistical equivalence relative to all measurements. (Recall that the assumption of preparation noncontextuality only has nontrivial consequences when two preparations are statistically equivalent for all measurements.)

The minimal assumption for our experiment would therefore be that the four measurements we perform are tomographically complete. But our physical understanding of the experiment leads us to a stronger assumption, that three measurements are tomographically complete. Here we clarify why, given this latter assumption, it is necessary to carry out the step of fitting to an appropriate GPT.

It is again easier to begin by considering the case that our experiment is described by quantum theory. Let $(q^1_{t,b}, q^2_{t,b}, q^3_{t,b}, q^4_{t,b})$ denote the probability of obtaining outcome '0' in measurements M_1, M_2, M_3, M_4 on preparation $P_{t,b}$, according to quantum theory, namely $q^i_{t,b} = \mathrm{Tr}(E_i \rho_{t,b})$, where E_i is the POVM element corresponding the the 0 outcome of measurement M_i and $\rho_{t,b}$ is the density operator for $P_{t,b}$.

Let us represent $\rho_{t,b} = \frac{1}{2}(\mathbb{I} + \boldsymbol{\sigma} \cdot \boldsymbol{u}_{t,b})$ by a Bloch vector $\boldsymbol{u}_{t,b}$ and the elements $E_i = v^0_i \mathbb{I} + \boldsymbol{\sigma} \cdot \boldsymbol{v}_i$ by a "Bloch four-vector" $(v^0_i, \boldsymbol{v}_i)$. Then $q^i_{t,b} = v^0_i + \boldsymbol{u}_{t,b} \cdot \boldsymbol{v}_i$. Since the $\boldsymbol{v}_i$ lie in a unit sphere, the $(q^1_{t,b}, q^2_{t,b}, q^3_{t,b}, q^4_{t,b})$ lie in the image of the sphere under the affine transformation $\boldsymbol{u} \mapsto (v^0_1, v^0_2, v^0_3, v^0_4) + (\boldsymbol{v}_1 \cdot \boldsymbol{u}, \boldsymbol{v}_2 \cdot \boldsymbol{u}, \boldsymbol{v}_3 \cdot \boldsymbol{u}, \boldsymbol{v}_4 \cdot \boldsymbol{u})$, i.e. some ellipsoid, a three-dimensional shape in a four-dimensional space.

However, the relative frequencies we observe will fluctuate from $q^i_{t,b}$ in all four dimensions. Fluctuations in the three dimensions spanned by the "Bloch ellipsoid" can be accommodated by using secondary preparations as described above. But fluctuations in the fourth direction are, according to quantum theory, always statistical and never systematic, and by the same token we cannot deliberately produce supplementary preparations that have any bias in this fourth direction. Therefore, we need to deal with these fluctuations in a different way. If one was assuming quantum theory, one would simply fit relative frequencies to the closest points $q^{t'}_{t,b}$ in the Bloch ellipsoid, just as one usually fits to the closest valid density operator.

Since we do not assume quantum theory, we do not assume that the states lie in an ellipsoid. However, we still make the assumption that three two-outcome measurements are tomographically complete. Hence, by Proposition 1, the long-run probabilities lie in a three-dimensional subspace of a four-dimensional space, and so there are no supplementary preparations that can deal with fluctuations of relative frequencies in the fourth dimension. Instead of fitting to the "Bloch ellipsoid", we fit to a suitable GPT.

Analysis of statistical errors

Because the relative frequencies derived from the raw data constitute a finite sample of the true probabilities (i.e. the long-run relative frequencies), the GPT states and effects that yield the best fit to the raw data are *estimates* of the GPT states and effects that characterize the primary preparations and measurements.

It is these estimates that we input into the linear program that identifies the weights with which the primary procedures must be mixed to yield secondary procedures. As such, our linear program outputs estimates of the true weights, and therefore when we use these weights in mixtures of our estimates of the primary GPT states and effects, we obtain estimates of the secondary GPT states and effects. In turn, these estimates are input into the expression for A and yield an estimate of the value of A for the secondary preparations and measurements.

To determine the statistical error on our estimate of A, we must quantify the statistical error on our estimates of the GPT states for the primary preparations and on our estimates of the GPT effects for the primary measurements. We do so by taking our experimental data in 100 distinct runs, each of which yields one such estimate. For each of these, we follow the algorithm for computing the value of A. In this way, we obtain 100 samples of the value of A for the secondary procedures, and these are used to determine the statistical error on our estimate for A.

Note that a different approach would be to presume some statistical noise model for our experiment, then input the observed relative frequencies (averaged over the entire experiment) into a program that adds noise using standard Monte Carlo techniques. Though one could generate a greater number of samples of A in this way, such an approach would be worse than the one we have adopted because the error analysis would be only as reliable as one's assumptions regarding the nature of the noise.

Given that the quantity A we obtain is 2300 σ above the noncontextual bound, we can conclude that there is a very low likelihood that a noncontextual model would provide a better fit to the true probabilities than the GPT that best fit our finite sample would. This is the sense in which our experiment rules out a noncontextual model with high confidence.

It should be noted that this sort of analysis of statistical errors is no different from that which has historically been used for experimental tests of Bell inequalities. The Bell quantity (the expression that is bounded in a Bell inequality) is defined in terms of the true probabilities. Any Bell experiment, however, only gathers a finite sample of these true probabilities. From this sample, one estimates the true probabilities and in turn the value of the Bell quantity. We treat the quantity A appearing in our noncontextuality inequality in a precisely analogous manner. The definition of A in terms of the true probabilities is admittedly more complicated than for a Bell quantity: we define secondary procedures based on an optimization problem that takes as input the true probabilities for the primary procedures, and use the true probabilites for the secondary procedures to define A. But this complication does not change the

fact that A is ultimately just a function of the true probabilities for the primary preparations and measurements, albeit a function that incorporates a particular linear optimization problem in its definition.

Recently, more sophisticated statistical techniques have been applied to the analysis of tests of Bell inequalities [14–19]. Specifically, one computes an upper bound on the probability that a locally causal model could reproduce the Bell quantity observed in the experiment. This techniques has been applied to the analysis of the recent loophole-free violations of Bell inequalities [20–22]. It would be worthwhile to make a similar analysis of our experiment, by computing an upper bound on the probability that a noncontextual model could reproduce the value of A we observe. Such an analysis is outside the scope of the present work, but an interesting problem for future work in this area.

SUPPLEMENTARY REFERENCES

[1] Spekkens, R. W. Contextuality for preparations, transformations, and unsharp measurements. *Phys. Rev. A* **71**, 052108 (2005).

[2] Bell, J. S. The theory of local beables. *Epistemological Lett.* **9**, 11–24 (1976). (reproduced in Dialectica **39**, 85 (1985)).

[3] Kochen, S. & Specker, E. The problem of hidden variables in quantum mechanics. *Indiana Univ. Math. J.* **17**, 59–87 (1968).

[4] Spekkens, R. W., Buzacott, D. H., Keehn, A. J., Toner, B. & Pryde, G. J. Preparation contextuality powers parity-oblivious multiplexing. *Phys. Rev. Lett.* **102**, 010401 (2009).

[5] Bell, J. S. On the Einstein-Podolsky-Rosen paradox. *Physics* **1**, 195–200 (1964).

[6] Clauser, J. F., Horne, M. A., Shimony, A. & Holt, R. A. Proposed experiment to test local hidden-variable theories. *Phys. Rev. Lett.* **23**, 880–884 (1969).

[7] Clauser, J. F. & Horne, M. A. Experimental consequences of objective local theories. *Phys. Rev. D* **10**, 526–535 (1974). URL `http://link.aps.org/doi/10.1103/PhysRevD.10.526`.

[8] Spekkens, R. W. The status of determinism in proofs of the impossibility of a noncontextual model of quantum theory. *Found. Phys.* **44**, 1125–1155 (2014).

[9] Harrigan, N. & Spekkens, R. W. Einstein, incompleteness, and the epistemic view of quantum states. *Found. Phys.* **40**, 125–157 (2010).

[10] Hardy, L. Quantum theory from five reasonable axioms. Preprint at http://arxiv.org/abs/quant-ph/0101012 (2001). (2001).

[11] Barrett, J. Information processing in generalized probabilistic theories. *Phys. Rev. A* **75**, 032304 (2007).

[12] Krystek, M. & Anton, M. A weighted total least-squares algorithm for fitting a straight line. *Meas. Sci. Technol.* **18**, 3438 (2007).

[13] Press, W. H., Teukolsky, S. A., Vetterling, W. T. & Flannery, B. P. *Numerical Recipes: The Art of Scientific Computing* (Cambridge University Press, New York, 2007), 3 edn.

[14] Gill, R. D. Accardi contra bell (cum mundi): The impossible coupling. *Lecture Notes-Monograph Series* **42**, 133–154 (2003). URL `http://www.jstor.org/stable/4356235`.

[15] Zhang, Y., Glancy, S. & Knill, E. Asymptotically optimal data analysis for rejecting local realism. *Phys. Rev. A* **84**, 062118 (2011). URL `http://link.aps.org/doi/10.1103/PhysRevA.84.062118`.

[16] Zhang, Y., Glancy, S. & Knill, E. Efficient quantification of experimental evidence against local realism. *Phys. Rev. A* **88**, 052119 (2013). URL `http://link.aps.org/doi/10.1103/PhysRevA.88.052119`.

[17] Bierhorst, P. A rigorous analysis of the Clauser-Horne-Shimony-Holt inequality experiment when trials need not be independent. *Foundations of Physics* **44**, 736–761 (2014). URL `http://dx.doi.org/10.1007/s10701-014-9811-3`.

[18] Bierhorst, P. A robust mathematical model for a loophole-free Clauser-Horne experiment. *Journal of Physics A: Mathematical and Theoretical* **48**, 195302 (2015). URL `http://stacks.iop.org/1751-8121/48/i=19/a=195302`.

[19] Kofler, J., Giustina, M., Larsson, J.-Å. & Mitchell, M. W. Requirements for a loophole-free photonic bell test using imperfect setting generators. Preprint at http://arxiv.org/abs/1411.4787 (2015). (2015).

[20] Hensen, B. *et al.* Loophole-free bell inequality violation using electron spins separated by 1.3 kilometres. *Nature* **526**, 682–686 (2015). URL `http://dx.doi.org/10.1038/nature15759`.

[21] Shalm, L. K. *et al.* Strong loophole-free test of local realism. *Phys. Rev. Lett.* **115**, 250402 (2015). URL `http://link.aps.org/doi/10.1103/PhysRevLett.115.250402`.

[22] Giustina, M. *et al.* Significant-loophole-free test of bell's theorem with entangled photons. *Phys. Rev. Lett.* **115**, 250401 (2015). URL `http://link.aps.org/doi/10.1103/PhysRevLett.115.250401`.
